# Stereoselective Inhibition of High- and Low-Affinity Organic Cation Transporters

**DOI:** 10.1021/acs.molpharmaceut.3c00691

**Published:** 2023-11-14

**Authors:** Lukas Gebauer, Ole Jensen, Muhammad Rafehi, Jürgen Brockmöller

**Affiliations:** Institute of Clinical Pharmacology, University Medical Center Göttingen, D-37075 Göttingen, Germany

**Keywords:** transporter inhibition, organic cation transporters, stereoselectivity, solute carrier, drug enantiomers

## Abstract

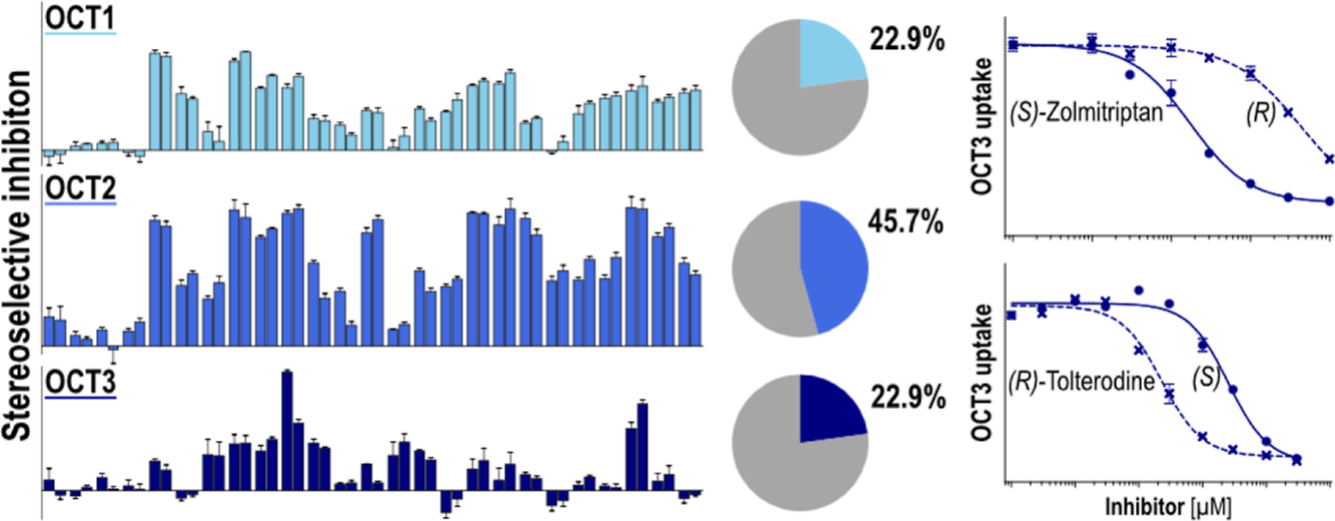

Many drugs have chiral centers and are therapeutically applied as racemates. Thus, the stereoselectivity in their interactions with membrane transporters needs to be addressed. Here, we studied stereoselectivity in inhibiting organic cation transporters (OCTs) 1, 2, and 3 and the high-affinity monoamine transporters (MATs) NET and SERT. Selectivity by the inhibition of 35 pairs of enantiomers significantly varied among the three closely related OCTs. OCT1 inhibition was nonselective in almost all cases, whereas OCT2 was stereoselectively inhibited by 45% of the analyzed drugs. However, the stereoselectivity of the OCT2 was only moderate with the highest selectivity observed for pramipexole. The (*R*)-enantiomer inhibited OCT2 4-fold more than the (*S*)-enantiomer. OCT3 showed the greatest stereoselectivity in its inhibition. (*R*)-Tolterodine and (*S*)-zolmitriptan inhibited OCT3 11-fold and 25-fold more than their respective counterparts. Interestingly, in most cases, the pharmacodynamically active enantiomer was also the stronger OCT inhibitor. In addition, stereoselectivity in the OCT inhibition appeared not to depend on the transported substrate. For high-affinity MATs, our data confirmed the stereoselective inhibition of NET and SERT by several antidepressants. However, the stereoselectivity measured here was generally lower than that reported in the literature. Unexpectedly, the high-affinity MATs were not significantly more stereoselectively inhibited than the polyspecific OCTs. Combining our in vitro OCT inhibition data with available stereoselective pharmacokinetic analyses revealed different risks of drug–drug interactions, especially at OCT2. For the tricyclic antidepressant doxepine, only the (*E*)-isomer showed an increased risk of drug–drug interactions according to guidelines from regulatory authorities for renal transporters. However, most chiral drugs show only minor stereoselectivity in the inhibition of OCTs in vitro, which is unlikely to translate into clinical consequences.

## Introduction

Relevant stereoselectivity is well known for several inhibitors of the high-affinity serotonin transporter (SERT, *SLC6A4*). The (*S*)-enantiomers of citalopram,^1^ duloxetine,^2^ and venlafaxine^3^ inhibit SERT significantly more potent than the (*R*)-enantiomers, and duloxetine was from the beginning approved as an enantiopure drug. For citalopram, a chiral switch was performed, and the (*S*)-enantiomer with the generic name escitalopram may have a better risk–benefit ratio than racemic citalopram.^4^ On the other hand, chiral switching was not always successful. Exemplarily, (*R*)-fluoxetine, (*S*)-fenfluramine, and (*S*)-sotalol had a worse outcome in clinical studies.^5−7^ The underlying mechanisms, not in all instances well understood, may rely on different off-target effects of the enantiomers; for instance, (*S*)-sotalol has none of the possibly beneficial beta-receptor blocking activity.

The polyspecific organic cation transporters (OCTs) of the SLC22A gene family are important for the absorption, distribution, and elimination of many drugs and other substances.^8^ OCT1 and OCT2 (*SLC22A1* and *−2*) are highly expressed in the liver and kidney,^9,10^ respectively, whereas OCT3 (*SLC22A3*) has no dominant primary tissue expression and can be found in cardiac tissue,^11^ in the brain,^12^ and at the blood–brain barrier.^13^ All three mediate the cellular uptake of numerous drugs but also of endogenous and environmental compounds.^14,15^

OCT inhibition has been studied in detail. Data for several hundred inhibitors of OCT1, 2, and 3 have been published.^13,16−18^ A higher molecular weight, higher number of rings, and increased lipophilicity have been identified as the most relevant features of the OCT inhibitors compared to non- or only weakly inhibiting substances. However, many studies neglected the aspect of ligand stereochemistry and simply studied racemates as inhibitors or only one enantiomer. This appears as a gap because most small-molecule drugs are chiral products clinically used as racemates.^19,20^ The enantiomers of many drugs differ significantly in their biological activity.^19,21^ Accordingly, stereospecific differences in the inhibition of membrane transporters could be another reason to prefer enantiopure substances over their racemic mixtures, assuming that enantiopure substances cause fewer drug–drug interactions (DDIs) in membrane transport.

An early study on membrane transport on the brush border membrane of opossum kidney (OK) cells identified stereoselective inhibition of TEA uptake by verapamil.^22^ However, the underlying transporters were not identified. Another study addressed the binding of propranolol and atenolol enantiomers to OCT1 using immobilized OCT1 as the liquid chromatography stationary phase,^23^ but only minor effects of stereoselectivity were observed. A consensus variant of OCT1 was stereoselectively inhibited by the enantiomer of the antiarrhythmic drug verapamil but this variant was characterized by a sequence identity of 87% toward the wild-type sequence.^24^ Another study of the inhibition of OCTs by central nervous system drugs revealed a twofold difference in the inhibition of OCT1 by the α-pyrrolidinovalerophenone enantiomers but no difference in OCT2.^25^ For OCT3, no comprehensive data on stereoselective inhibition are available so far.

**Figure 1:**
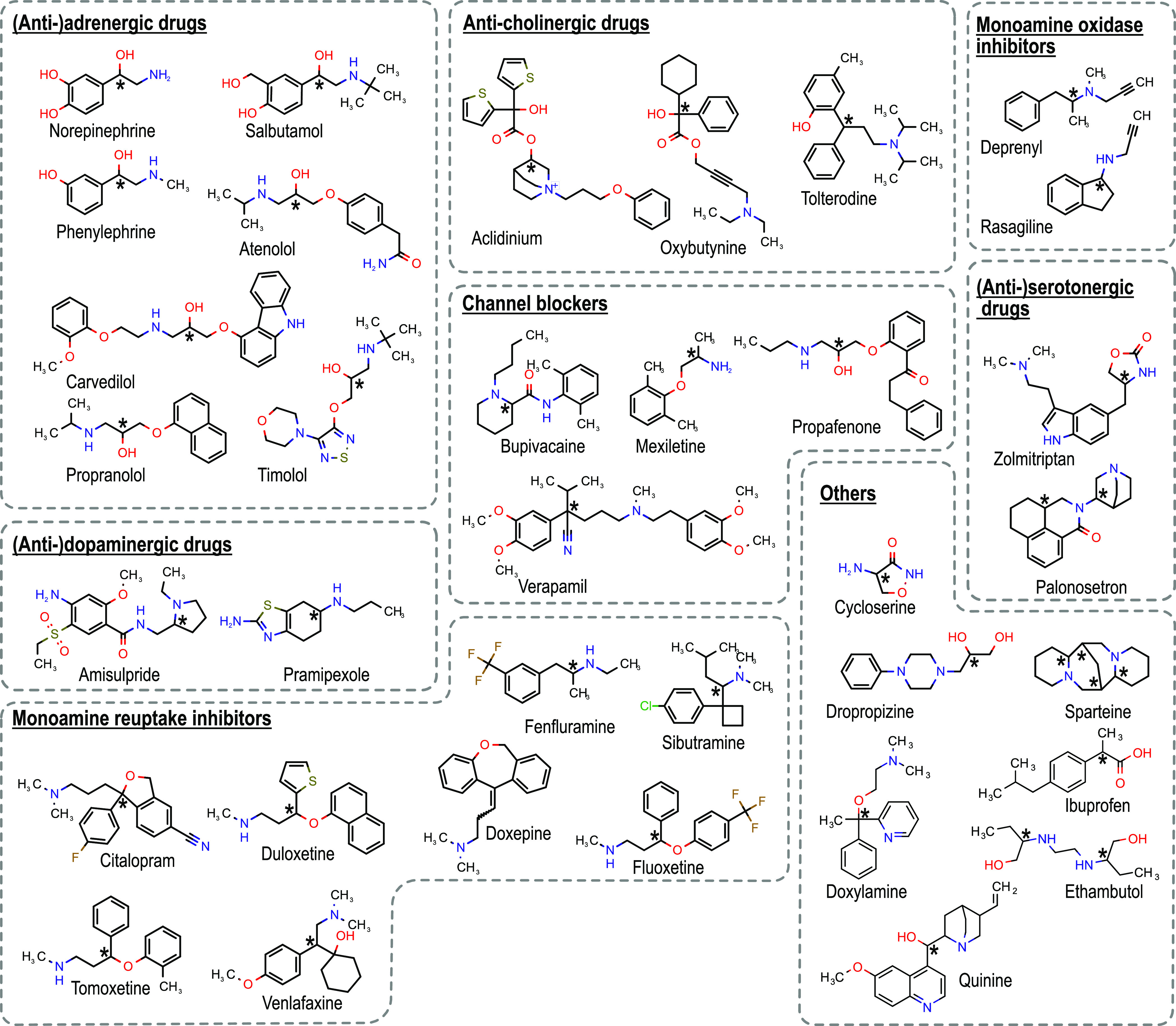
Structure of the investigated chiral drugs. Substances are classified according to their drug classes and chiral centers are highlighted with asterisk (*). Doxepine represents the particular case of (*E*) and (*Z*) stereoisomerism.

Here, we characterized the possible stereoselectivity in the inhibition of OCTs. We searched for chiral inhibitors based on previously published data.^12,15−17^ We then studied selected inhibitors, where both enantiomers were commercially available. In total, we analyzed 71 stereoisomers of 35 chiral substances being known or presumed inhibitors of OCTs (Figure 1). For screening, we used different transporter model substrates. We then characterized those enantiomers with relevant differences in their inhibition by concentration-dependent inhibition experiments. Moreover, we characterized the monoamine reuptake inhibitors in our test set for the stereoselective inhibition of monoamine transporters (MATs). Finally, we combined the extent of stereoselectivity in the OCT inhibition with available stereoselective pharmacokinetic data. By considering stereoselective blood concentrations and plasma protein binding, we estimated the potential of in vivo DDIs based on our in vitro inhibition data. This analysis might help to identify drugs where a chiral switch might result in a less interaction-causing drug.

## Experimental Section

### Test Compounds

Test compounds were purchased from HelloBio (Dunshaughlin, Republic of Ireland), Roche Pharma (Tübingen, Germany), Santa Cruz Biotechnology (Dallas, USA), Sigma-Aldrich (Darmstadt, Germany), and Toronto Research Chemicals (Toronto, Canada) with purities of at least 95%. A complete list of all substances, including the respective manufacturer and their catalog number, is provided in Table S1.

### In Vitro Characterization of Model Substrates

All experiments were carried out in transporter-overexpressing HEK293 cells. OCT1-, OCT2-, NET-, DAT-, and SERT-overexpressing cells as well as the empty-vector transfected control cell line were generated as described earlier,^26−28^ whereas the OCT3-overexpressing HEK293 cells were generous gift of from Drs. Koepsell and Gorbulev (University of Würzburg, Germany). Amino acid sequences of overexpressed transporters are given in Figure S1. All cells were cultivated in Dulbecco’s modified Eagle’s medium (DMEM) supplemented with 10% (v/v) FCS, penicillin (100 U/mL), and streptomycin (100 μg/mL) and kept in culture for no longer than 30 passages.

Prior to inhibition studies, the used model substrates were validated in concentration-dependent uptake experiments if no literature data were available (Table S2 and Figures S2 and S3). Thereby, we ensured that the model substrate concentration used in the subsequent inhibition experiments was sufficiently below the *K*_m_ value of its uptake kinetics. For the uptake experiments, 300,000 HEK293 cells were plated in poly-d-lysine-coated 24-well plates 48 h ahead of the experiments. On the day of the experiment, the cells were washed once with prewarmed to 37 °C HBSS (Thermo Fisher Scientific, Darmstadt, Germany) supplemented with 10 mM HEPES and pH adjusted to 7.4 (Sigma-Aldrich)—hereafter termed HBSS+. For concentration-dependent experiments, the cells were incubated with increasing substrate concentrations for 2 min. Uptake was terminated by adding ice-cold HBSS+. Afterward, the cells were washed twice with ice-cold HBSS+, and cell lysis was done with 80% (v/v) acetonitrile (LGC Standards, Wesel, Germany) to which an internal standard for eventual LC–MS/MS analysis had been added. Intracellularly accumulated substrates were then quantified by LC–MS/MS analysis, and calibration was performed with known concentrations of the respective substances. All uptake data were normalized to the number of seeded cells by measuring the total protein content in radioimmunoprecipitation assay buffer-lysed cells in a bicinchoninic acid assay.^29^

### In Vitro Inhibition Experiments

As for the transport experiments, 300,000 HEK293 cells were plated in poly-d-lysine-coated 24-well plates 2 days ahead of the experiment. On every plate, two wells of empty vector (EV)-transfected cells were used as a control to account for the passive influx of the model substrate, and two wells of transporter-overexpressing cells were used to determine the noninhibited uptake of the model substrate. Cells were initially washed once with 37 °C HBSS+ and then incubated with 2 μM model substrate (0.2 μM for MPP^+^ uptake by MATs) with and without 20 μM inhibitor for 5 min. For concentration-dependent inhibition experiments, the cells were incubated with increasing inhibitor concentrations. The incubation was terminated by aspiration of the buffer before cells were washed twice with ice-cold HBSS+ and lysed in 80% acetonitrile. Cell lysates were transferred into a black 96-well plate for the fluorescence measurement of ASP^+^ or used for sample preparation for LC–MS/MS analysis. ASP^+^ was quantified using a Tecan Ultra (Tecan, Crailsheim, Germany) multiplate reader using an excitation wavelength of 482 nm and an emission wavelength of 612 nm. Fluorescence quantification was performed in technical duplicates.

### Concentration Analyses by LC–MS/MS

Intracellular drug concentrations of nonfluorescent substrates were quantified by liquid chromatography coupled to mass spectrometry (LS–MS/MS). The HPLC was a Shimadzu Nexera HPLC system (formed by an autosampler SIL-30AC, a column oven CTO-20AC, a pump LC-39AD, and a controller CBM-20A, all from Shimadzu, Kyoto, Japan). Chromatography was carried out on a Brownlee SPP RP-Amide column (4.6 × 100 mm inner dimension with 2.7 μm particle size, PerkinElmer, Waltham, MA) with a C18 precolumn. The aqueous mobile phase contained 0.1% (v/v) formic acid and organic additive [acetonitrile/methanol (6:1), both LGC Standards] ranging from 3 to 20% to adjust the retention times of the analytes. Separation was done at a flow rate of 0.3 or 0.4 mL/min with a column oven temperature of 40 °C. Compound detection was performed with an API 4000 tandem mass spectrometer (AB SCIEX, Darmstadt, Germany) operating in multiple reaction monitoring mode. Analyte peaks were integrated using Analyst software (version 1.6.2, AB SCIEX). A complete list of MS detection parameters and the mobile phase compositions is provided in Table S3.

### Calculations

Transporter-mediated net uptake was calculated as the difference between uptake in transporter-overexpressing cells and that in the empty-vector-transfected control. Transport kinetic parameters were determined by nonlinear regression following the Michaelis–Menten equation (*v* = *v*_max_ × [*S*]/(*K*_m_ + [*S*])) using GraphPad Prism (Version 5.01 for Windows, GraphPad Software, La Jolla, CA, USA). *V*_max_ is the maximum transport velocity, while *K*_m_ is defined as the substance concentration that is required to reach half of *v*_max_. The ratio of *v*_max_ over *K*_m_ is termed intrinsic clearance Cl_int_. For inhibition experiments, the transport activity was calculated by using the following equation
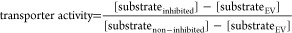


Substrate_EV_ refers to the passive uptake of model substrates into the control cells and substrate_noninhibited_ is the uptake in transporter-overexpressing cells without the presence of any inhibitor. The percent inhibition values are then calculated as described in the following
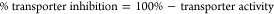


For concentration inhibition experiments, the transporter activity was plotted against the log_10_ of inhibitor concentrations, and the data were fitted by the following equation to determine IC_50_ values
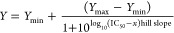


*Y* refers to the transporter activity, whereas *Y*_max_ is the maximum and *Y*_min_ is the minimal transporter activity. X is the log_10_ of inhibitor concentrations, IC_50_ is the half-maximal inhibitory concentration, and hill slope is the slope factor. The regression was done using GraphPad Prism (Version 5.01 for Windows, GraphPad Software).

## Results

The investigated inhibitors included known OCT substrates such as salbutamol, sparteine, and zolmitriptan as well as substances previously identified as not-transported inhibitors like tolterodine and verapamil.^15^ Except for ibuprofen, all substances were positively charged at physiological pH and had at least one chiral center.

### Stereoselective Inhibition of OCT1, OCT2, and OCT3

**Figure 2:**
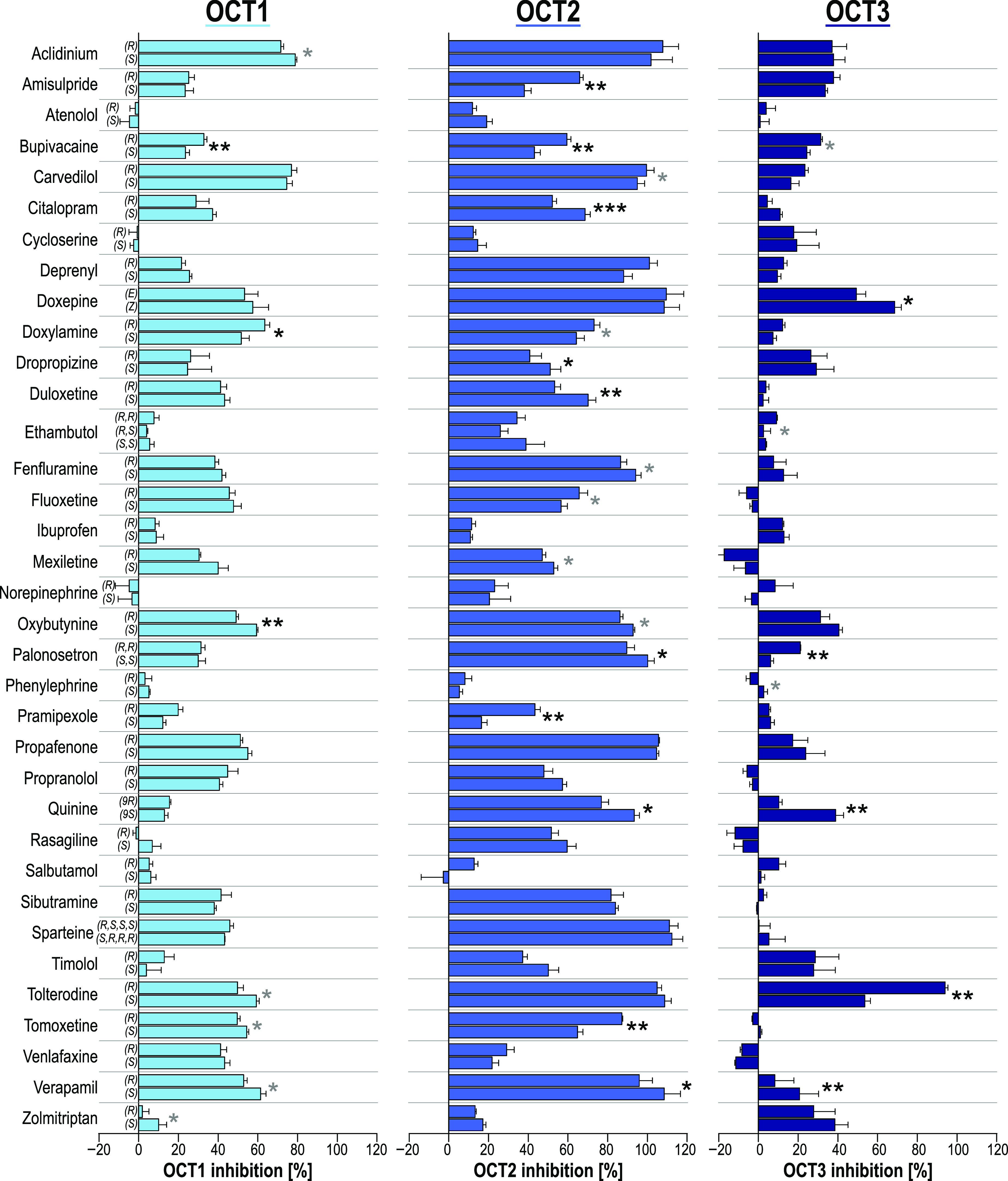
Screening of OCT inhibition using ASP^+^ as the fluorescent model substrate. HEK293 cells overexpressing OCT1–3 were incubated with 2 μM ASP^+^ in the presence or absence of 20 μM inhibitor for 5 min. Empty-vector-transfected cells were used as a control to account for passive diffusion. Data are presented as percentage inhibition values with mean ± SEM of three independent experiments. Asterisks indicate the statistical significance of the differences between the two enantiomers (Student’s *t* test; *p < 0.05, **p < 0.01, ***p < 0.001). Enantiomeric differences greater than 10 percentage points are shown in black asterisks, and the differences below are shown in gray.

Initially, we tested 35 pairs of stereoisomers for differences in their levels of inhibition of the OCT using fluorescent ASP^+^ as a model substrate (Figure 2).

**Figure 3:**
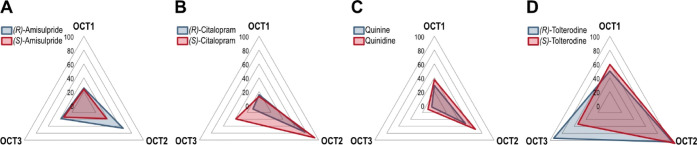
Chiral OCT inhibition spider plots showing mean values of ASP^+^ percentage inhibition of OCT1, 2, and 3 by enantiomers of amisulpride (A), citalopram (B), quinine/quinidine (C), and tolterodine (D).

When applying only statistical significance as the criterion, 8, 16, and 8 substances showed stereoselective inhibition of OCT1, −2, and −3, respectively. However, the differences in the level of the inhibition of the OCT among most enantiomers were relatively small. When considering only those effects as relevant where the difference between the inhibitions by both enantiomers is at least 10 percentage points, only 3, 10, and 5 stereoselective inhibitors were identified, respectively. OCT1 inhibition was characterized by almost no stereoselectivity, while numerous drugs stereoselectively inhibited OCT2. The enantiomers of the antipsychotic drug amisulpride, the selective serotonin reuptake inhibitor citalopram, and the antimalarial drug quinine showed noticeable differences in the inhibition of OCT2 (Figure 3A–C). Tolterodine enantiomers showed the highest stereoselectivity in this screening. (*R*)-Tolterodine showed a 94% inhibition of OCT3, whereas the corresponding (*S*)-enantiomer inhibited OCT3 only to 54% at our screening concentration (Figure 3D).

OCT2-mediated ASP^+^ uptake was inhibited most strongly by the inhibitors tested here, and several substances showed total transporter inhibition at the used concentration. Since complete transporter inhibition could mask any effects of stereoselectivity, we further analyzed enantiomeric pairs with an inhibition greater than 90% with reduced inhibitor concentrations (Figure S4). This revealed additionally relevant stereoselectivity in the inhibition of the OCT2 by duloxetine, fenfluramine, and palonosetron. In contrast to OCT2, OCT3-mediated ASP^+^ uptake was generally most weakly inhibited by the investigated substances (Figure 2).

**Figure 4:**
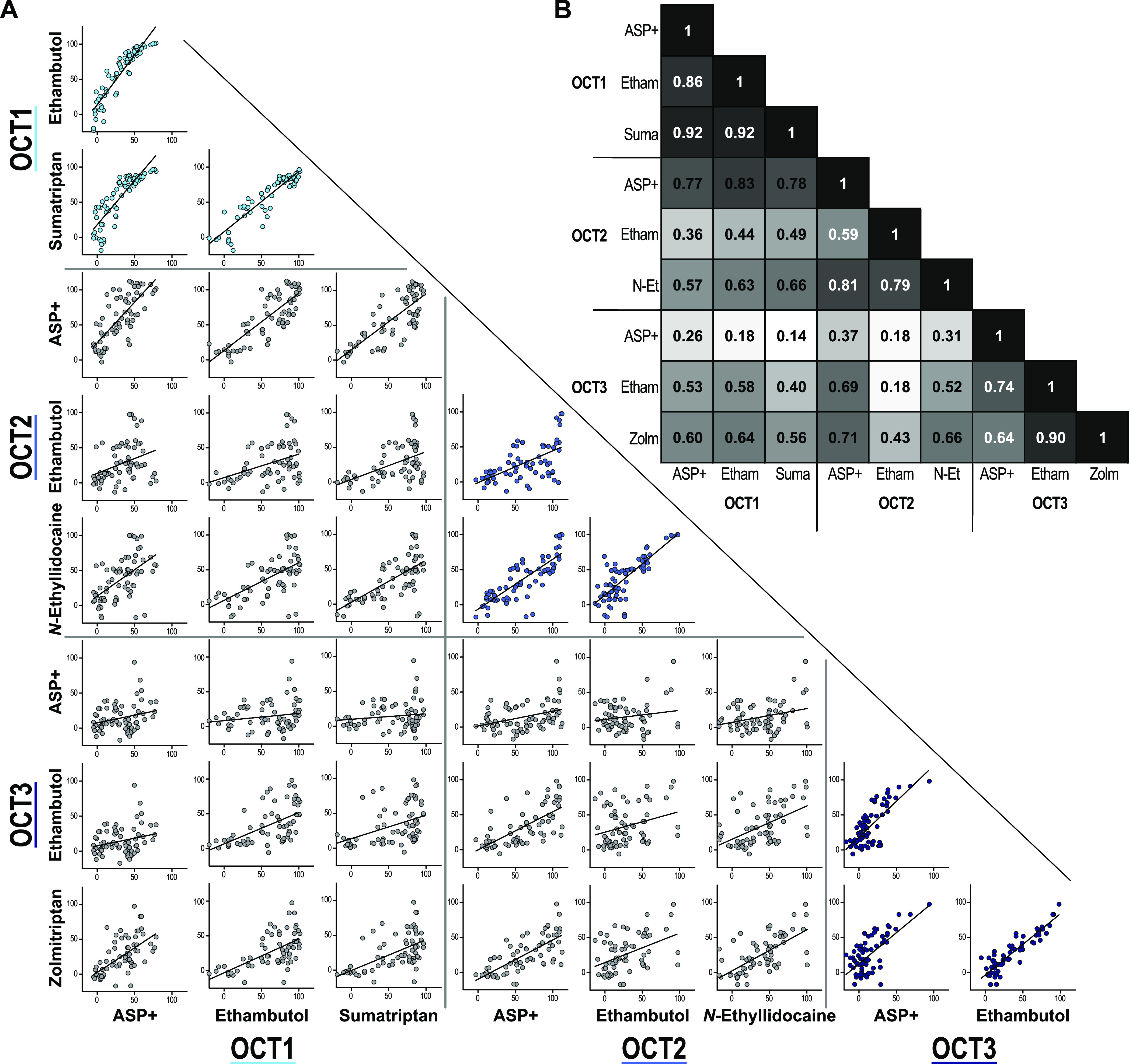
Correlation of OCT inhibition data for different model substrates. OCT inhibition was tested using (*S*,*S*)-ethambutol as a model substrate for OCT1–3 and sumatriptan, *N*-ethyllidocaine, and (*S*)-zolmitriptan as specific substrates for OCT1, −2, and −3, respectively (A). Mean values of three independent experiments were correlated. Correlation coefficients are shown in (B).

To elucidate whether stereoselectivity in the inhibition of the OCT is specific for the transported substrate, we tested all substances for the inhibition of the uptake of other OCT substrates. We used (*S*,*S*)-ethambutol for all three OCTs and sumatriptan, *N*-ethyllidocaine, and (*S*)-zolmitriptan as rather specific substrates for OCT1, −2, and −3, respectively (Table S4). Generally, there was reasonable consistency with the results obtained for ASP^+^ (Figure 4). Especially, the inhibition of the OCT1 model substrates was highly correlated.

For OCT3, the inhibition of ethambutol and zolmitriptan was higher as compared with the inhibition of the ASP^+^ uptake. As the inhibition of ASP^+^ transport was often too weak to observe any effects of stereoselectivity at the tested concentration, comparison to the other substrates revealed stereoselective inhibition of OCT3 by bupivacaine, oxybutynine, propranolol, verapamil, and zolmitriptan.

**Figure 5:**
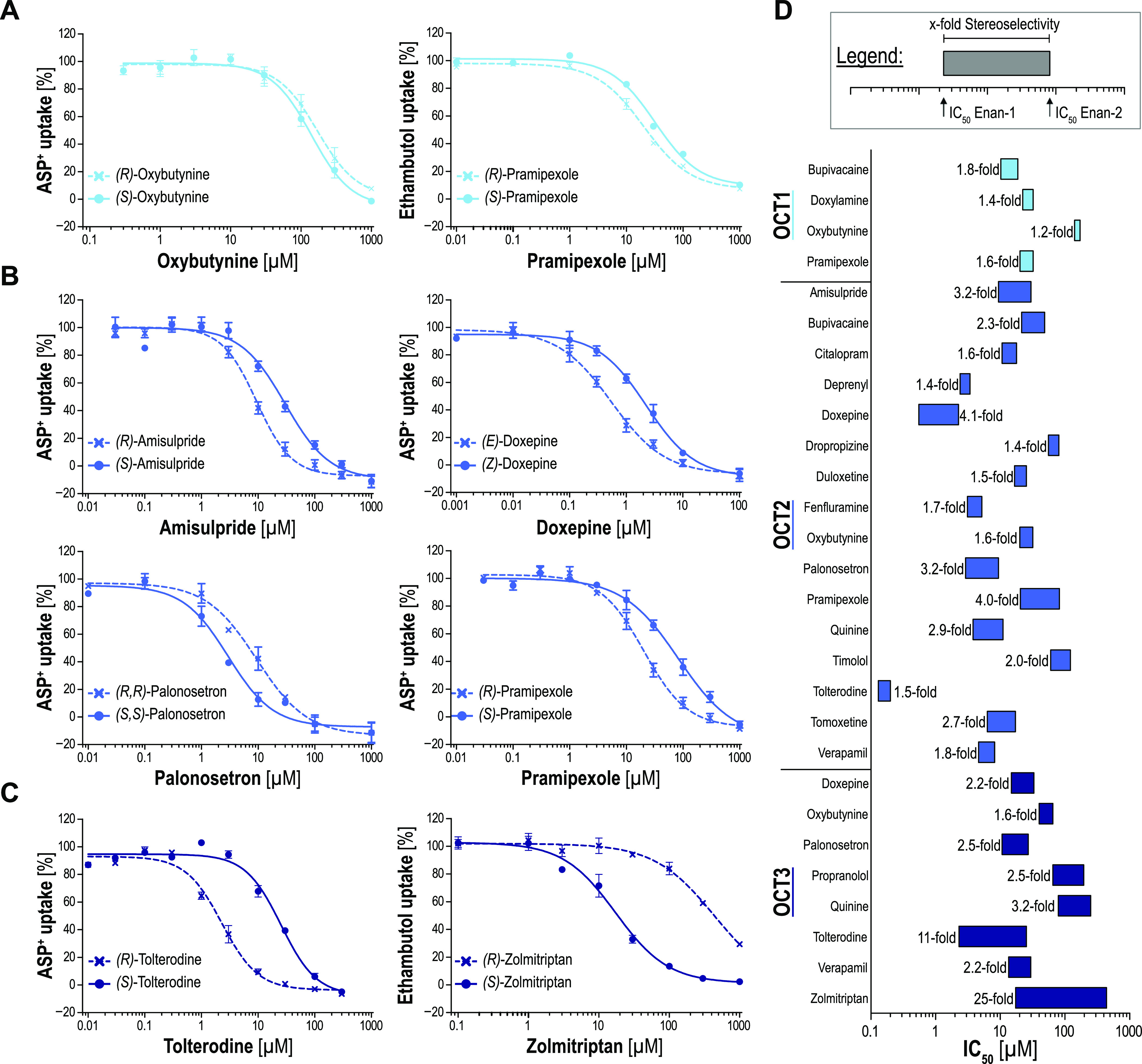
Stereoselective inhibition kinetics of OCT1, 2, and 3: OCT1 (A), OCT2 (B), and OCT3 (C) overexpressing HEK293 cells were incubated with 2 μM ASP^+^ or ethambutol with increasing inhibitor concentrations for 5 min. Empty-vector-transfected control cells were used as a control for no transporter-mediated uptake of the model substrate. Overview of concentration-dependent inhibition data (D). The bar length represents the stereoselectivity of chiral OCT inhibitors as each end represents the IC_50_ value of one enantiomer.

The enantiomers that showed relevant differences in their inhibitory effects were further characterized by concentration-dependent analyses (Figure 5, Figures S5–S7). ASP^+^ was used as a model substrate where possible. However, when the screening results showed low inhibition of ASP^+^ uptake and thus indicated that high inhibitor concentrations might be required to depict the full inhibition curve, ethambutol was used as a model substrate instead.

The observed stereoselectivity ratios ranged from 1.2-fold for bupivacaine enantiomers in the inhibition of the OCT1 up to 25-fold for zolmitriptan enantiomers and the inhibition of the OCT3, respectively (Table S5). In line with the screening results, stereoselectivity in OCT1 inhibition was very low. Most pairs of enantiomers were tested for stereoselective OCT2 inhibition. The selectivity ratios ranged from 1.5-fold for tolterodine enantiomers up to 4.0-fold for the enantiomers of pramipexole. The highest selectivities were observed for OCT3. (*R*)-tolterodine was 11-fold more potent in the inhibition of OCT3 and (*S*)-zolmitriptan even 25-fold more potent than their respective counterparts.

**Figure 6:**
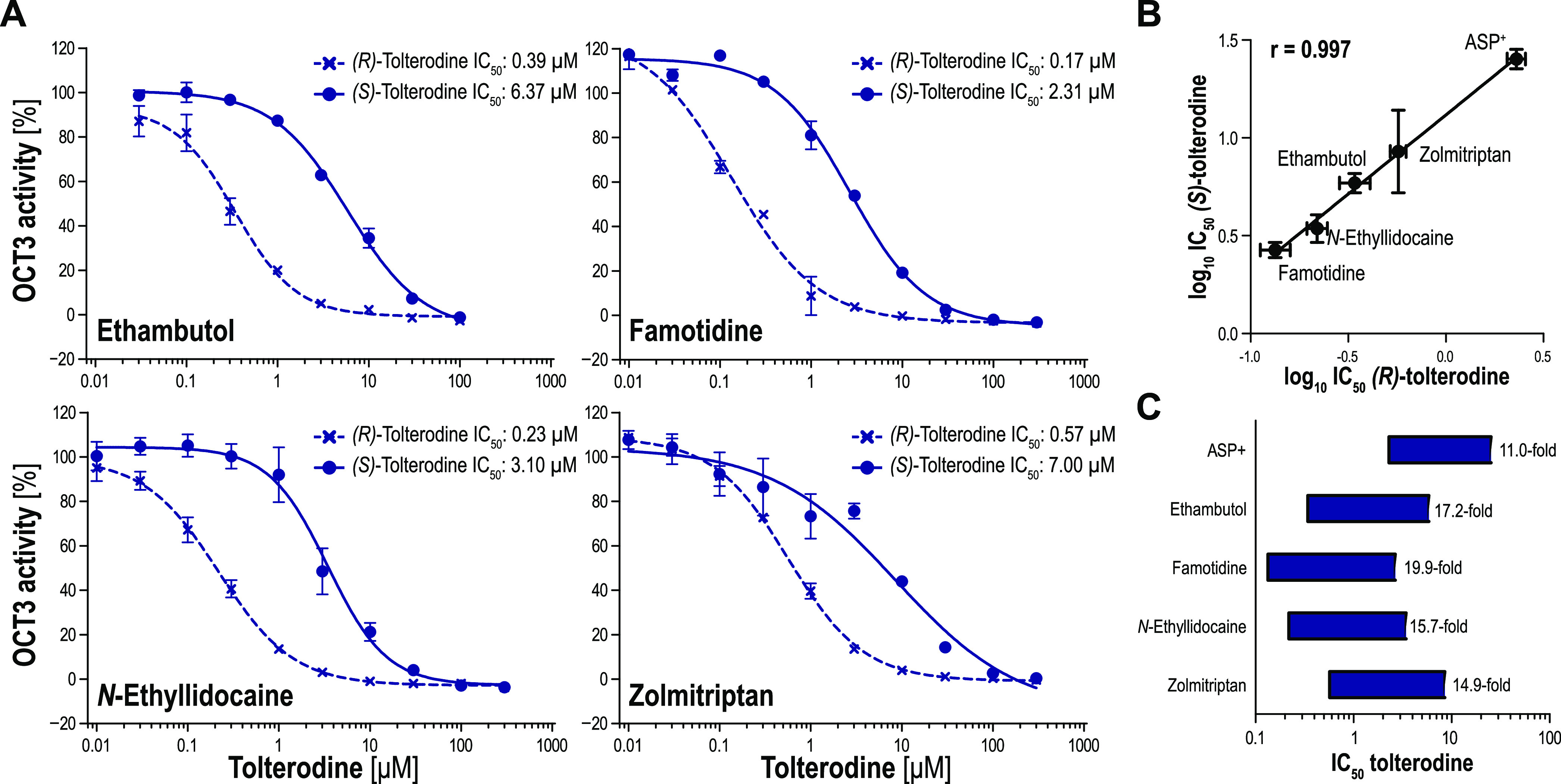
Correlation of OCT3 inhibition by tolterodine enantiomers for different model substrates. OCT3-overexpressing HEK293 cells were incubated with 2 μM (*S*,*S*)-ethambutol, famotidine, *N*-ethyllidocaine, and (*S*)-zolmitriptan with or without increasing concentrations of tolterodine enantiomers for 5 min (A). Intracellular accumulated model substrates were then quantified by LC–MS/MS analysis. Empty-vector-transfected control cells were used as the control to account for the unspecific influx of the model substrates by passive diffusion. Data are presented as mean ± SEM of three independent experiments. Correlation of obtained IC_50_ values for tolterodine enantiomers for the five substrates tested (B). Stereoselectivity in the inhibition of OCT3 is illustrated by the length of the bars as each end represents the IC_50_ value of one enantiomer (C).

As the tolterodine enantiomers showed high stereoselectivity and high affinity toward OCT3, both were tested for the uptake inhibition of further OCT3 substrates. The uptake of ethambutol, famotidine, *N*-ethyllidocaine, and zolmitriptan was inhibited more strongly as compared to ASP^+^ by the tolterodine enantiomers (Figure 6A). Nevertheless, there was an almost perfect correlation between the inhibition by both enantiomers (*r* = 0.99, Figure 6B). Also, the stereoselectivity in the inhibition of all five transported substrates was highly similar, although stereoselectivity was higher for the four newly tested substrates compared to ASP^+^ (Figure 6C).

### Comparison to High-Affinity Monoamine Transporters

**Figure 7:**
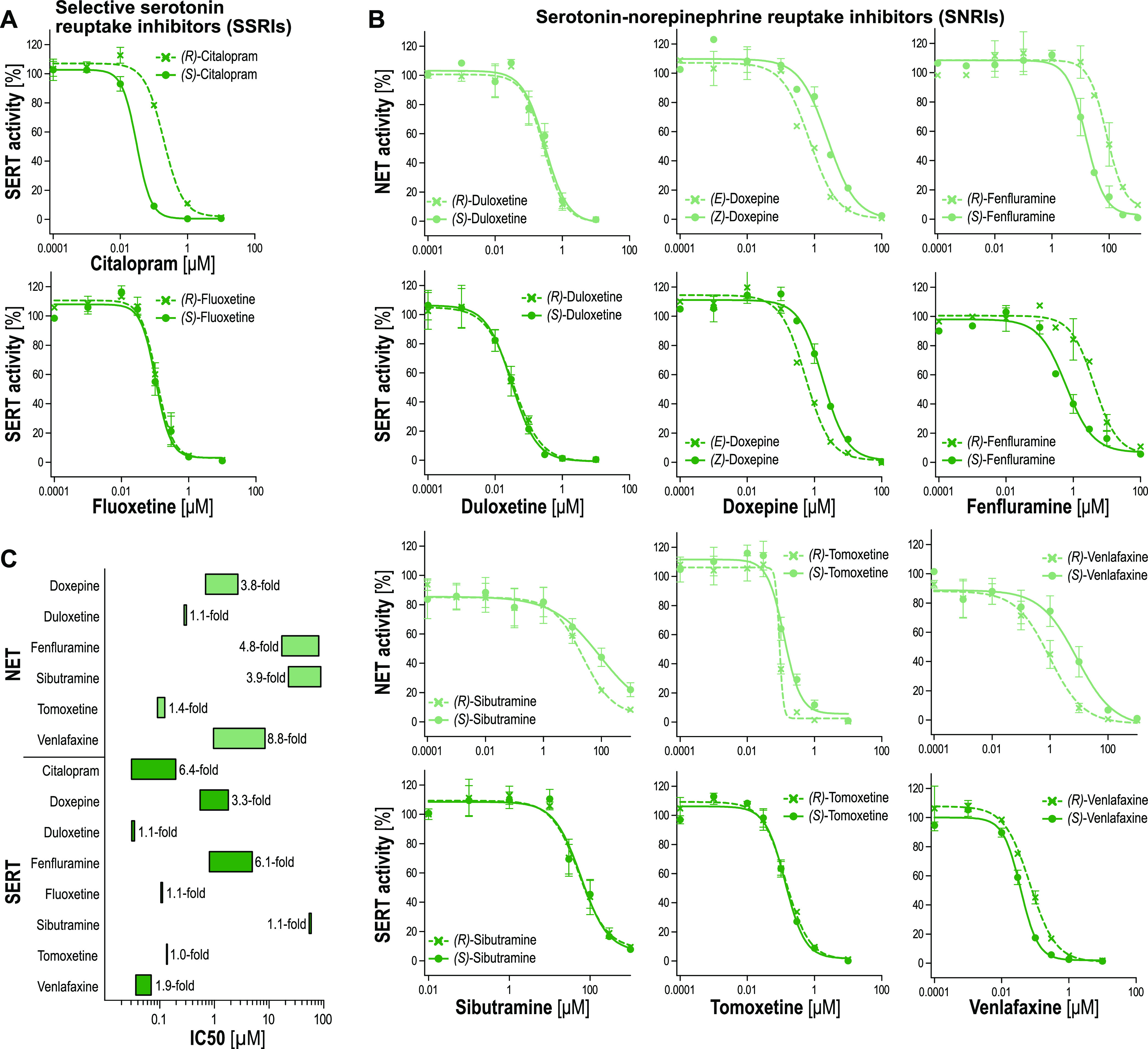
Stereoselective inhibition of the norepinephrine transporter and serotonin reuptake transporter by SSRIs and SNRIs. NET- and SERT-overexpressing cells were incubated with 0.2 μM MPP^+^ for 5 min in the presence or absence of increasing concentrations of SSRIs (A) and SNRI (B). Empty-vector-transfected control cells were incubated simultaneously to account for nonspecific MPP^+^ uptake. Subsequently, cells were lysed after washing, and intracellularly accumulated MPP^+^ was quantified by LC–MS/MS analysis. Data are presented as mean ± SEM of three independent experiments. Stereoselectivity in NET and SERT inhibition is illustrated by the length of the bars, as each end represents the IC_50_ value of one enantiomer (C).

A large class of the investigated substances were drugs used as monoamine reuptake inhibitors. For a comparative analysis, we tested these substances for their inhibition of MATs, as well. Since all monoamine reuptake inhibitors available here as pairs of the two enantiomers were either classified as selective serotonin reuptake inhibitors (SSRIs) or as serotonin-norepinephrine reuptake inhibitors (SNRI), we tested these drugs only for SERT or for NET and SERT inhibition (Figure 7, Table S6).

Among the SSRIs, (*S*)-citalopram showed a sixfold more potent SERT inhibition compared to the corresponding (*R*)-enantiomer. In contrast, fluoxetine enantiomers showed identical inhibitory potencies. NET and SERT were inhibited more strongly by the (*E*)-isomer of doxepine, with selectivity ratios of 3.8- and 3.3-fold, respectively. Also a similar stereoselectivity was also observed for the fenfluramine isomers. (*R*)-Fenfluramine was more potent in inhibiting NET and SERT, with selectivity ratios of 4.8- and 6.1-fold. However, both fenfluramine enantiomers were approximately 20-fold more potent in SERT inhibition than in NET inhibition. Interestingly, venlafaxine enantiomers showed the opposite stereoselectivity in NET and SERT inhibition. (*S*)-Venlafaxine had an 8.8-fold higher IC_50_ value for NET inhibition than the (*R*)-enantiomer, whereas (*R*)-venlafaxine was only slightly (selectivity ratio of 1.9) more potent in SERT inhibition. Similar to fenfluramine, the venlafaxine enantiomers were also more potent as SERT inhibitors than as NET inhibitors.

## Discussion

Here, we tested numerous chiral inhibitors from different drug classes for stereoselective differences of OCT inhibition. In contrast to drug membrane transporters, stereoselectivity is a well-studied feature in receptor binding and drug metabolism.^30−32^

Overall, stereoselectivity did not appear as a general feature of OCT inhibition, although certain drug–transporter pairs showed relevant differences in the inhibition between their enantiomers. However, this cannot be generalized since we observed remarkable differences between the three closely related transporters. Especially, OCT1 showed almost no relevant stereoselective inhibition. In contrast, OCT2 was inhibited stereoselectively by 45% of the tested drugs, although only to a moderate extent. For OCT3, we observed the strongest effects of stereoselectivity in this study. OCT3 was inhibited 11-fold stronger by the (*R*)-enantiomer of tolterodine than the corresponding (*S*)-enantiomer. Additionally, zolmitriptan enantiomers differed in their level of inhibition of OCT3 by 25-fold. However, tolterodine is marked as l-tartrate salt of the single (*R*)-enantiomer, and zolmitriptan is also used in the enantiopure form as (*S*)-enantiomer. Accordingly, high stereoselectivity in transporter inhibition has no direct clinical consequences with these two drugs. Among the stereoselective OCT2 inhibitors were many drugs used as monoamine reuptake inhibitors such as citalopram, doxepine, and tomoxetine. Interestingly, the enantiomers more active at MATs also inhibited OCT2 more strongly than their respective counterparts.

The trend of higher stereoselectivity in OCT2 and −3 inhibition compared to OCT1 aligns with previous studies on stereoselective OCT transport demonstrating that OCT2 and OCT3 act more stereoselectively than OCT1.^33,34^ Recent structural data based on cryogenic electron microscopy (cryo-EM) have confirmed a fundamentally similar substrate binding cavities of all three OCTs.^24,35^ Nevertheless, there were also more than 10 amino acids that differ between the three transporters and may therefore be responsible for substrate- and stereoselectivity. The long-known polyspecificity of OCTs^36^ was reflected for OCT1 by an orthosteric and opportunistic ligand binding site.^24^ However, without extensive site-directed mutagenesis driven by the newly available structures, any explanation of the differences in stereoselective OCT inhibition remains speculative. Additional cryo-EM structures with bound zolmitriptan or tolterodine enantiomers might be interesting. However, the moderate differences in inhibitory potencies for most drugs do not guarantee success in the identification of amino acids responsible for the stereoselectivity.

To estimate whether the observed effects of stereoselectivity could lead to stereoselective DDIs in vivo, we compared our data to available data on stereoselective pharmacokinetics. For this, we combined enantiospecific plasma concentrations with protein binding whenever this was available (Table 1). However, for protein binding, we rarely found data on stereoselective binding and thus had to refer to protein binding data that did not reflect stereochemistry. According to guidelines provided by the European Medicines Agency (EMA), a drug is concerned regarding DDIs at renal uptake transporters, for instance, OCT2, if the unbound maximal plasma concentrations are greater than 0.02 times the determined IC_50_ or *K*_i_ values (*I*_max, u_/IC_50_ ≥ 0.02). The U.S. Food and Drug Administration (FDA) applies a more liberal cutoff of 0.1 for the risk of renal interactions at OCT2.

**Table 1:** Estimation of the Risk of Stereoselective In Vivo Drug–Drug Interactions^a^

transporter	drug	IC_50_ [μM]	study design	*I*_max_ [μM]	*f*_u,p_	risk score [*I*_max, u_/IC_50_]
OCT1	(*R*)-bupivacaine	10.2	single dose (30 mg), intravenous administration^37^	0.228	0.07^37^	0.005
	(*S*)-bupivacaine	18.7		0.154	0.05^37^	0.003
OCT2	(*R*)-amisulpride	9.4	single dose (50 mg), oral administration^38^	0.158	0.84^39^	0.014
	(*S*)-amisulpride	29.8		0.182		0.005
	(*R*)-bupivacaine	21.4	single dose (30 mg), intravenous administration^37^	0.228	0.07^37^	0.003
	(*S*)-bupivacaine	48.4		0.154	0.05^37^	0.001
	(*R*)-citalopram	17.8	multiple doses (40 mg, once daily for 21 d), oral administration^40^	0.228	0.20^41^	0.003
	(*S*)-citalopram	10.7		0.154		0.003
	(*E*)-doxepine	0.55	single dose (75 mg), oral administration^42^	0.286	0.25^43^	**0.130**
	(*Z*)-doxepine	2.26		0.039		0.004
	(*R*)-verapamil	8.2	multiple doses (80 mg, thrice daily for 7d), oral administration^44^	0.519	0.05^45^	0.003
	(*S*)-verapamil	4.6		0.216	0.08^45^	0.004
OCT3	(*E*)-doxepine	33.0	single dose (75 mg), oral administration^42^	0.286	0.25^43^	0.002
	(*Z*)-doxepine	14.9		0.039		<0.001
	(*R*)-propranolol	195	single dose (80 mg), oral administration^46^	0.228	0.25^47^	<0.001
	(*S*)-propranolol	69		0.154	0.20^47^	<0.001

a *C*_max_: maximum plasma concentration. *f*_u,p_: fraction unbound in plasma. *I*_max_: mean steady state *C*_max_ (*I*_max_ was replaced by *C*_max_ when no stereoselective steady-state pharmacokinetic data were available). *I*_max,u_: unbound maximal plasma concentrations.

Although most of the studied chiral drugs did not reach a risk score above the EMA- or FDA-suggested thresholds, the estimated *I*_max,u_/IC_50_ ratios showed considerable differences between the enantiomers of several drugs. Not reaching the thresholds is partially caused by the fact that stereoselectivity is not always considered in pharmacokinetic studies, and therefore, the stereospecific pharmacokinetic parameters are not available. For amisulpride, several pharmacokinetic studies are available with higher dosing up to 1200 mg and accordingly also higher plasma concentrations but without stereoselective analyses.^48^ Using these plasma concentrations for drug–drug risk estimation would result in scores above the EMA or FDA threshold. Another consideration is the impact of the model substrate used for transporter inhibition. As shown exemplarily for the tolterodine enantiomers (Figure 6), the relative transporter inhibition was almost perfectly correlated for different substrates. Nevertheless, there were significant differences in absolute terms. OCT3 uptake of famotidine was inhibited with 10-fold higher potencies by tolterodine enantiomers than ASP^+^ uptake via OCT3. This indicates that for a more reliable estimation of transporter-mediated DDIs, specific combinations of drugs, which are likely to be administered together, should be analyzed. The potential victim drug should be used as the model substrate and the perpetrator drug as the inhibitor.

Interestingly, in most cases where we observed stereoselectivity in the OCT inhibition, the pharmacodynamically active stereoisomer inhibited the transporters most strongly. With doxepine, the pharmacodynamically active (*E*)-stereoisomer showed a higher inhibition of OCT2 and a strongly increased risk of DDI compared to the (*Z*)-stereoisomer. Apparently, (*E*)-doxepine at OCT2 was the only drug that would raise concerns regarding DDIs according to the EMA or FDA guidelines in our study. In contrast to that of OCT2, OCT3 was inhibited more strongly by the (*Z*)-stereoisomer of doxepine. Due to an 85:15 formulation of (*E*)- versus (*Z*)-doxepine, the higher plasma levels resulted also in a higher risk of DDI at the OCT3 by (*E*)-doxepine compared to (*Z*)-doxepine. Nevertheless, based on the available pharmacokinetic data, the risk score did not reach the EMA threshold for both stereoisomers. Significant stereoselectivity was also described for the metabolism of doxepine, and (*E*)-doxepine biotransformation was shown to be highly dependent on the highly polymorphic CYP2D6.^42^ Accordingly, individuals with intermediate or poor metabolizer genotypes might be at a significantly increased risk of (*E*)-doxepine-mediated DDIs.

Norepinephrine, norphenylephrine, and salbutamol were among those drugs for which a stereoselective OCT transport was previously identified.^33,34^ However, for none of those drugs, a relative stereoselectivity in the inhibition of OCTs was observed. This might be largely due to the fact that these were only weak inhibitors, which is in line with previous observations that substrates are generally only weak inhibitors.^15,18^ Thus, higher drug concentrations could reveal potential stereoselectivity in their inhibition, although this would then be of limited relevance. For other transported substrates such as zolmitriptan, stereoselective transport has not been studied yet. Given the high stereoselectivity in the inhibition of OCT3 by zomitriptan enantiomers, this might be a promising characterization.

Stereoselectivity in the inhibition of MATs has been studied already in detail.^1−3^ The stereoselectivities observed here were generally in line with previous studies. However, the extent of selectivity was lower than that reported in the literature, although the direction of stereoselectivity was the same. Exemplarily, for citalopram, a stereoselectivity of 125-fold was reported initially,^1^ which is much higher than the sixfold selectivity observed in our system. In the initial report, uptake inhibition of radiolabeled serotonin into rat brain synaptosomes was analyzed, whereas we used human SERT-overexpressing HEK293 cells and MPP^+^ as the model substrate. These methodologic differences could explain the differences in the observed stereoselectivities. However, other studies reported stereoselectivities of 40-fold^49^ or even only 26-fold^50^ in SERT inhibition by both citalopram enantiomers. Both are still higher than the stereoselectivity we have observed here, but this nevertheless illustrates that the differences in inhibitory potencies of citalopram enantiomers vary also in other studies. With fluoxetine, the lack of stereoselectivity we observed in SERT inhibition by fluoxetine enantiomers is in agreement with the literature data.^51^ Also, a higher potency of (*S*)-fenfluramine over that of its counterpart could be confirmed in our system.

## Conclusions

Altogether, the extent of stereoselectivity in OCT inhibition varied greatly, although all three transporters are closely related. This further substantiates that stereoselectivity is a highly transporter-specific property. Consequently, all transporters potentially exposed to DDIs by chiral drugs should be characterized individually, regarding possible effects of stereoselectivity in their inhibition. The highly similar inhibitory potencies of most chiral substances in particular at OCT1 could still be seen as a further reason to prefer enantiopure formulations over their racemic mixtures in drug therapy. Since for most chiral drugs, the intended bioactivity mainly relies on one enantiomer, the other enantiomer might be indeed considered as “isomeric ballast”^52^ in the context of OCT inhibition. Therefore, using enantiopure formulations might result in a higher selectivity as a result of a reduced potential of DDIs at OCTs.

## Supporting Information Available

Test substances including drug SMILES, manufacturer, and catalog number; transport kinetic parameters of used model substrates; mass spectrometry detection parameters and HPLC mobile phase composition; OCT1, 2, and 3 inhibition screening data; IC_50_ values of stereoselective OCT inhibition; IC_50_ values of stereoselective MAT inhibition; amino acid sequences of overexpressed OCTs and MATs; MPP^+^ transport by monoamine transporters; novel transport data for OCT model substrates; inhibition screening of OCT2 ASP^+^ uptake with reduced inhibitor concentrations; stereoselective concentration-dependent OCT1 inhibition; stereoselective concentration-dependent OCT2 inhibition; and stereoselective concentration-dependent OCT3 inhibition (PDF)

## Abbreviations

ASP^+^: 4-(4-(dimethylamino)styryl)-*N*-methylpyridinium
Cryo-EM: cryogenic electron microscopy
DAT: dopamine transporter
DDIs: drug–drug interactions
DMEM: Dulbecco’s modified Eagle’s medium
EMA: European Medicines Agency
FDA: US Food and Drug Administration
HPLC: high-pressure liquid chromatography
IPA: isopropyl alcohol
LC–MS/MS: liquid chromatography coupled to tandem mass spectrometry
MAT: monoamine transporter
MPP^+^: 1-methyl-4-phenylpyridinium
NET: norepinephrine transporter
OCT: organic cation transporter
SEM: standard error of the mean
SERT: serotonin transporter
SMILES: simplified molecular input line entry specification
SNRI: serotonin-norepinephrine reuptake inhibitor
SSRI: selective serotonin reuptake inhibitor
