# Supplementary material for: Stereoselective Inhibition of High- and Low-Affinity Organic Cation Transporters
Source: Mol Pharm. 2023 Nov 14;20(12):6289–300. doi: 10.1021/acs.molpharmaceut.3c00691 (PMC10698726; doi:10.1021/acs.molpharmaceut.3c00691)
Supplement: Supplementary file 1 — mp3c00691_si_001.pdf [file mp3c00691_si_001.pdf]

Supporting information

# Stereoselective Inhibition of High- and Low affinity Organic Cation Transporters

Lukas Gebauer*, Ole Jensen, Muhammad Rafehi, and Jürgen Brockmöller

Institute of Clinical Pharmacology, University Medical Center Göttingen, D-37075 Göttingen, Germany

E-Mail: lukas.gebauer@med.uni-goettingen.de

**Table of contents**

Table S1: Test substances including drug SMILES, manufacturer, and catalogue number

Table S2: Transport kinetic parameters of used model substrates

Table S3: Mass spectrometry detection parameters and HPLC mobile phase composition

Table S4: OCT1, 2, and 3 inhibition screening data

Table S5: $IC_{50}$ values of stereoselective OCT inhibiton

Table S6: $IC_{50}$ values of stereoselective MAT inhibition

Figure S1: Amino acid sequences of overexpressed OCTs and MATs

Figure S2: Novel transport data for OCT model substrates

Figure S3: $MPP^+$ transport by monoamine transporters

Figure S4: Inhibition screening of OCT2 $ASP^+$ uptake with reduced inhibitor concentrations

Figure S5: Stereoselective concentration-dependent OCT1 inhibition

Figure S6: Stereoselective concentration-dependent OCT2 inhibition

Figure S7: Stereoselective concentration-dependent OCT3 inhibition

**Table S1: Test substances including drug SMILES, manufacturer, and catalogue number**

| Substance | Isomeric SMILES | Manufacturer | Cat# |
|---|---|---|---|
| *(R)*-Aclidinium | C1C[N+]2(CCC1[C@H](C2)OC(=O)C(C3=CC=CS3)(C4=CC=CS4)O)CCCOC5=CC=CC=C5 | Sigma[a] | SML2868 |
| *(S)*-Aclidinium | C1C[N+]2(CCC1[C@@H](C2)OC(=O)C(C3=CC=CS3)(C4=CC=CS4)O)CCCOC5=CC=CC=C5 | TRC[b] | A190190 |
| *(R)*-Amisulpride | CCN1CCC[C@@H]1CNC(=O)C2=CC(=C(C=C2OC)N)S(=O)(=O)CC | TRC[b] | A633255 |
| *(S)*-Amisulpride | CCN1CCC[C@H]1CNC(=O)C2=CC(=C(C=C2OC)N)S(=O)(=O)CC | TRC[b] | A633260 |
| *(R)*-Atenolol | CC(C)NC[C@H](COC1=CC=C(C=C1)CC(=O)N)O | Sigma[a] | 330884 |
| *(S)*-Atenolol | CC(C)NC[C@@H](COC1=CC=C(C=C1)CC(=O)N)O | Sigma[a] | A143 |
| *(R)*-Bupivacaine | CCCCN1CCCC[C@@H]1C(=O)NC2=C(C=CC=C2C)C | TRC[b] | B689545 |
| *(S)*-Bupivacaine | CCCCN1CCCC[C@H]1C(=O)NC2=C(C=CC=C2C)C | TRC[b] | B689675 |
| *(R)*-Carvedilol | COC1=CC=CC=C1OCCNC[C@H](COC2=CC=CC3=C2C4=CC=CC=C4N3)O | Roche[e] | 12040190 |
| *(S)*-Carvedilol | COC1=CC=CC=C1OCCNC[C@@H](COC2=CC=CC3=C2C4=CC=CC=C4N3)O | Roche[e] | 12040189 |
| *(R)*-Citalopram | CN(C)CCC[C@]1(C2=C(CO1)C=C(C=C2)C#N)C3=CC=C(C=C3)F | SCBT[c] | sc-219751 |
| *(S)*-Citalopram | CN(C)CCC[C@@]1(C2=C(CO1)C=C(C=C2)C#N)C3=CC=C(C=C3)F | Sigma[a] | PHR1733 |
| *(R)*-Cycloserine | C1[C@H](C(=O)NO1)N | Sigma[a] | C6880 |
| *(S)*-Cycloserine | C1[C@@H](C(=O)NO1)N | Sigma[a] | C1159 |
| *(R)*-Deprenyl | C[C@H](CC1=CC=CC=C1)N(C)CC#C | Sigma[a] | M003 |
| *(S)*-Deprenyl | C[C@@H](CC1=CC=CC=C1)N(C)CC#C | TRC[b] | D288595 |
| *(E)*-Doxepine | CN(C)CC/C=C/1\C2=CC=CC=C2COC3=CC=CC=C31 | TRC[b] | D550005 |
| *(Z)*-Doxepine | CN(C)CC/C=C\1/C2=CC=CC=C2COC3=CC=CC=C31 | TRC[b] | D550015 |
| *(R)*-Doxylamine | C[C@@](C1=CC=CC=C1)(C2=CC=CC=N2)OCCN(C)C | TRC[b] | D561995 |
| *(S)*-Doxylamine | C[C@](C1=CC=CC=C1)(C2=CC=CC=N2)OCCN(C)C | TRC[b] | D562035 |
| *(R)*-Dropropizine | C1CN(CCN1C[C@H](CO)O)C2=CC=CC=C2 | Sigma[a] | L0420002 |
| *(S)*-Dropropizine | C1CN(CCN1C[C@@H](CO)O)C2=CC=CC=C2 | Sigma[a] | SML2511 |
| *(R)*-Duloxetine | CNCC[C@H](C1=CC=CS1)OC2=CC=CC3=CC=CC=C32 | TRC[b] | D720990 |
| *(S)*-Duloxetine | CNCC[C@@H](C1=CC=CS1)OC2=CC=CC3=CC=CC=C32 | HB[d] | HB1806 |
| *(R,R)*-Ethambutol | CC[C@H](CO)NCCN[C@H](CC)CO | TRC[b] | E889805 |
| *(R,S)*-Ethambutol | CC[C@H](CO)NCCN[C@@H](CC)CO | TRC[b] | E67805 |
| *(S,S)*-Ethambutol | CC[C@@H](CO)NCCN[C@@H](CC)CO | Sigma[a] | E4630 |
| *(R)*-Fenfluramine | CCN[C@H](C)CC1=CC(=CC=C1)C(F)(F)F | TRC[b] | F247595 |

| | | | |
|---|---|---|---|
| *(S)*-Fenfluramine | CCN[C@@H](C)CC1=CC(=CC=C1)C(F)(F)F | TRC[b] | F247596 |
| *(R)*-Fluoxetine | CNCC[C@H](C1=CC=CC=C1)OC2=CC=C(C=C2)C(F)(F)F | Sigma[a] | F1678 |
| *(S)*-Fluoxetine | CNCC[C@@H](C1=CC=CC=C1)OC2=CC=C(C=C2)C(F)(F)F | Sigma[a] | F1553 |
| *(R)*-Ibuprofen | C[C@H](C1=CC=C(C=C1)CC(C)C)C(=O)O | TRC[b] | I140005 |
| *(S)*-Ibuprofen | C[C@@H](C1=CC=C(C=C1)CC(C)C)C(=O)O | Sigma[a] | 375160 |
| *(R)*-Mexiletine | CC1=C(C(=CC=C1)C)OC[C@@H](C)N | TRC[b] | M340790 |
| *(S)*-Mexiletine | CC1=C(C(=CC=C1)C)OC[C@H](C)N | TRC[b] | M340795 |
| *(R)*-Norepinephrine | C1=CC(=C(C=C1[C@H](CN)O)O)O | SCBT[c] | sc357366 |
| *(S)*-Norepinephrine | C1=CC(=C(C=C1[C@@H](CN)O)O)O | Sigma[a] | 392480 |
| *(R)*-Oxybutynine | CCN(CC)CC#CCOC(=O)[C@@](C1CCCCC1)(C2=CC=CC=C2)O | TRC[b] | O868510 |
| *(S)*-Oxybutynine | CCN(CC)CC#CCOC(=O)[C@](C1CCCCC1)(C2=CC=CC=C2)O | TRC[b] | O868515 |
| *(R,R)*-Palonosetron | C1C[C@H]2CN(C(=O)C3=CC=CC(=C23)C1)[C@H]4CN5CCC4CC5 | TRC[b] | P165805 |
| *(S,S)*-Palonosetron | C1C[C@@H]2CN(C(=O)C3=CC=CC(=C23)C1)[C@@H]4CN5CCC4CC5 | TRC[b] | P165800 |
| *(R)*-Phenylephrine | CNC[C@@H](C1=CC(=CC=C1)O)O | Sigma[a] | P6126 |
| *(S)*-Phenylephrine | CNC[C@H](C1=CC(=CC=C1)O)O | TRC[b] | P320630 |
| *(R)*-Pramipexole | CCCN[C@@H]1CCC2=C(C1)SC(=N2)N | TRC[b] | P700745 |
| *(S)*-Pramipexole | CCCN[C@H]1CCC2=C(C1)SC(=N2)N | TRC[b] | P700755 |
| *(R)*-Propafenone | CCCNC[C@H](COC1=CC=CC=C1C(=O)CCC2=CC=CC=C2)O | TRC[b] | P757495 |
| *(S)*-Propafenone | CCCNC[C@@H](COC1=CC=CC=C1C(=O)CCC2=CC=CC=C2)O | TRC[b] | P757496 |
| *(R)*-Propranolol | CC(C)NC[C@H](COC1=CC=CC2=CC=CC=C21)O | Sigma[a] | P0689 |
| *(S)*-Propranolol | CC(C)NC[C@@H](COC1=CC=CC2=CC=CC=C21)O | Sigma[a] | P8688 |
| Quinine | COC1=CC2=C(C=CN=C2C=C1)[C@H]([C@@H]3C[C@@H]4CCN3C[C@@H]4C=C)O | Sigma[a] | Q1125 |
| Quinidine | COC1=CC2=C(C=CN=C2C=C1)[C@@H]([C@H]3C[C@@H]4CCN3C[C@@H]4C=C)O | Sigma[a] | Q0750 |
| *(R)*-Rasagiline | C#CCN[C@@H]1CCC2=CC=CC=C12 | Sigma[a] | SML0124 |
| *(S)*-Rasagiline | C#CCN[C@H]1CCC2=CC=CC=C12 | TRC[b] | R126005 |
| *(R)*-Salbutamol | CC(C)(C)NC[C@@H](C1=CC(=C(C=C1)O)CO)O | TRC[b] | A514485 |
| *(S)*-Salbutamol | CC(C)(C)NC[C@H](C1=CC(=C(C=C1)O)CO)O | TRC[b] | A514490 |
| *(R)*-Sibutramine | CC(C)C[C@H](C1(CCC1)C2=CC=C(C=C2)Cl)N(C)C | TRC[b] | S422501 |
| *(S)*-Sibutramine | CC(C)C[C@@H](C1(CCC1)C2=CC=C(C=C2)Cl)N(C)C | TRC[b] | S422504 |
| (-)-Sparteine | C1CCN2C[C@@H]3C[C@H]([C@H]2C1)CN4[C@H]3CCCC4 | Sigma[a] | S2251 |
| (+)-Sparteine | C1CCN2C[C@H]3C[C@@H]([C@H]2C1)CN4[C@H]3CCCC4 | Sigma[a] | 92052 |
| *(R)*-Timolol | CC(C)(C)NC[C@H](COC1=NSN=C1N2CCOCC2)O | Sigma[a] | Y0000644 |

| | | | |
|---|---|---|---|
| *(S)*-Timolol | CC(C)(C)NC[C@@H](COC1=NSN=C1N2CCOCC2)O | Sigma[a] | T6394 |
| *(R)*-Tolterodine | CC1=CC(=C(C=C1)O)C(CCN(C(C)C)C(C)C)C2=CC=CC=C2 | Sigma[a] | PZ0009 |
| *(S)*-Tolterodine | CC1=CC(=C(C=C1)O)[C@@H](CCN(C(C)C)C(C)C)C2=CC=CC=C2 | TRC[b] | T535796 |
| *(R)*-Venlafaxine | CN(C)C[C@@H](C1=CC=C(C=C1)OC)C2(CCCCC2)O | TRC[b] | V120003 |
| *(S)*-Venlafaxine | CN(C)C[C@H](C1=CC=C(C=C1)OC)C2(CCCCC2)O | TRC[b] | V120008 |
| *(R)*-Verapamil | CC(C)[C@@](CCCN(C)CCC1=CC(=C(C=C1)OC)OC)(C#N)C2=CC(=C(C=C2)OC)OC | Sigma[a] | V106 |
| *(S)*-Verapamil | CC(C)[C@](CCCN(C)CCC1=CC(=C(C=C1)OC)OC)(C#N)C2=CC(=C(C=C2)OC)OC | Sigma[a] | V105 |
| *(R)*-Zolmitriptan | CN(C)CCC1=CNC2=C1C=C(C=C2)C[C@@H]3COC(=O)N3 | Sigma[a] | Y0001986 |
| *(S)*-Zolmitriptan | CN(C)CCC1=CNC2=C1C=C(C=C2)C[C@H]3COC(=O)N3 | Sigma[a] | SML0248 |

[a] Sigma-Aldrich Chemie GmbH, Darmstadt, Germany
[b] Toronto Research Chemicals, Toronto, Canada
[c] Santa-Cruz Biotechnology, Dallas, USA
[d] HelloBio, Dunshaughlin, Republic of Ireland
[e] Roche Pharma, Tübingen, Germany

**Table S2: Transport kinetic parameters of used model substrates**

| Transporter | Substance | $K_m$ ± SEM [μM] | | | $v_{max}$ ± SEM [pmol × mg Protein$^{-1}$ × min$^{-1}$] | | | $Cl_{int}$ ± SEM | | | Reference |
|---|---|---|---|---|---|---|---|---|---|---|---|
| OCT1 | $ASP^+$ | 11.7 | ± | 1.6 | 2678 | ± | 71 | 230 | ± | 37 | this study |
| | *(S,S)*-Ethambutol | 167 | ± | 11 | 2351 | ± | 44 | 14.1 | ± | 1.2 | this study |
| | rac Fenoterol | 1.8 | ± | 0.2 | 57.2 | ± | 4.8 | 33.0 | ± | 2.3 | [1] |
| | Sumatriptan | 55.4 | ± | 7.8 | 978 | ± | 132 | 17.6 | | | [2] |
| OCT2 | $ASP^+$ | 29.6 | ± | 4.0 | 1812 | ± | 73 | 61.3 | ± | 10.8 | this study |
| | *(S,S)*-Ethambutol | 163 | ± | 59 | 5967 | ± | 645 | 36.6 | ± | 17.3 | this study |
| | *N*-Ethyllidocaine | 87.7 | ± | 17.0 | 5172 | ± | 247 | 58.9 | ± | 14.2 | this study |
| OCT3 | ASP+ | 16.5 | ± | 2.2 | 3381 | ± | 94 | 205 | ± | 33 | this study |
| | *(S,S)*-Ethambutol | 146 | ± | 34 | 2463 | ± | 164 | 16.9 | ± | 5.1 | this study |
| | Famotidine | 8.91 | ± | 3.43 | 242 | ± | 16 | 27.2 | ± | 7.3 | [3] |
| | *N*-Ethyllidocaine | 46.3 | ± | 17.8 | 407 | ± | 34 | 8.80 | ± | 4.12 | [3] |
| | *(S)*-Zolmitriptan | 24.8 | ± | 9.7 | 594 | ± | 56 | 24.0 | ± | 11.7 | this stud |
| NET | $MPP^+$ | 2.50 | ± | 0.97 | 381 | ± | 21 | 152 | ± | 68 | this study |
| DAT | $MPP^+$ | 20.1 | ± | 7.2 | 2107 | ± | 215 | 104 | ± | 47 | this study |
| SERT | $MPP^+$ | 21.1 | ± | 2.2 | 627 | ± | 17 | 29.7 | ± | 4.1 | this study |


1. Tzvetkov, M. V.; Matthaei, J.; Pojar, S.; Faltraco, F.; Vogler, S.; Prukop, T.; Seitz, T.; Brockmöller, J. Increased Systemic Exposure and Stronger Cardiovascular and Metabolic Adverse Reactions to Fenoterol in Individuals with Heritable OCT1 Deficiency. *Clinical Pharmacology & Therapeutics* **2018,** 103, 868-878.

2. Matthaei, J.; Kuron, D.; Faltraco, F.; Knoch, T.; Dos Santos Pereira, J. N.; Abu Abed, M.; Prukop, T.; Brockmöller, J.; Tzvetkov, M. V. OCT1 mediates hepatic uptake of sumatriptan and loss-of-function OCT1 polymorphisms affect sumatriptan pharmacokinetics. *Clinical pharmacology and therapeutics* **2016,** 99, 633-641.

3. Gebauer, L.; Jensen, O.; Brockmöller, J.; Dücker, C. Substrates and Inhibitors of the Organic Cation Transporter 3 and Comparison with OCT1 and OCT2. *Journal of Medicinal Chemistry* **2022,** 65, 12403-12416.

**Table S3: Mass spectrometry detection parameters and HPLC mobile phase composition**

| Compound | RT [min] | Mass Q1 [Da] | Mass Q3 [Da] | DP [V] | CE [V] | CXP [V] | Internal standard |
|---|---|---|---|---|---|---|---|
| **3% organic additive** (96.9% H2O, 0.1 % formic acid, 2.6% acetonitrile, 0.4% methanol) | | | | | | | |
| Choline-d9 | 2.6 | 113.1 | 69.1 (66.1) | 66 | 27 (44) | 12 (12) | - |
| *(S,S)*-Ethambutol | 2.5 | 205.2 | 116.1 (55.1) | 66 | 21 (45) | 6 (10) | Choline-d9 |
| **8% organic additive** (91.9% H2O, 0.1 % formic acid, 6.9% acetonitrile, 1.1% methanol) | | | | | | | |
| Famotidine | 4.4 | 338.5 | 189.0 (155.0) | 54 | 27 (43) | 12 (10) | Ranitidine-d6 |
| Ranitidine-d6 | 4.4 | 321.2 | 176.0 (130.1) | 65 | 25 (35) | 15 (15) | - |
| **20% organic additive** (79.9% H2O, 0.1 % formic acid, 17.2% acetonitrile, 2.8% methanol) | | | | | | | |
| Fenoterol | 3.5 | 310.3 | 109.1 (141.0) | 70 | 40 (26) | 12 (12) | Fenoterol-d6 |
| Fenoterol-d6 | 3.5 | 310.3 | 109.1 (141.0) | 70 | 40 (26) | 12 (12) | - |
| $MPP^+$ | 3.5 | 170.016 | 128.1 (102.2) | 100 | 42 (63) | 8 (6) | Fenoterol-d6 |
| *N*-Ethyllidocaine | 4.5 | 264.2 | 86.0 (58.0) | 81 | 36 (64) | 16 (11) | Fenoterol-d6 |
| Sumatriptan | 3.5 | 296.2 | 58.2 (251.2) | 50 | 30 (24) | 12 (12) | Fenoterol-d6 |
| Zolmitriptan | 3.4 | 288 | 243 (182) | 75 | 24 (35) | 12 (12) | Fenoterol-d6 |

## Table S4: OCT1, 2, and 3 inhibition screening data

| OCT1 | | | | | | | | | |
|---|---|---|---|---|---|---|---|---|---|
| **Drug** | **ASP+ inhibition [%] ± SEM** | | | ***(S,S)*-Ethambutol inhibition [%] ± SEM** | | | **Sumatriptan inhibition [%] ± SEM** | | |
| *(R)*-Aclidinium | 71.6 | ± | 1.1 | 98.8 | ± | 0.4 | 93.6 | ± | 0.4 |
| *(S)*-Aclidinium | 78.8 | ± | 0.6 | 101.4 | ± | 1.2 | 93.8 | ± | 1.2 |
| *(R)*-Amisulpride | 25.2 | ± | 2.3 | 57.7 | ± | 3.0 | 29.3 | ± | 3.0 |
| *(S)*-Amisulpride | 23.5 | ± | 3.4 | 51.7 | ± | 2.7 | 15.2 | ± | 2.7 |
| *(R)*-Atenolol | -1.7 | ± | 2.2 | -7.3 | ± | 3.2 | -0.9 | ± | 3.2 |
| *(S)*-Atenolol | -4.6 | ± | 3.9 | -23.0 | ± | 21.0 | 4.3 | ± | 21.0 |
| *(R)*-Bupivacaine | 32.8 | ± | 1.3 | 63.8 | ± | 2.4 | 59.2 | ± | 2.4 |
| *(S)*-Bupivacaine | 23.5 | ± | 1.7 | 50.6 | ± | 2.9 | 43.9 | ± | 2.9 |
| *(R)*-Carvedilol | 76.9 | ± | 2.2 | 100.5 | ± | 0.1 | 96.6 | ± | 0.1 |
| *(S)*-Carvedilol | 74.5 | ± | 2.3 | 100.4 | ± | 0.4 | 96.5 | ± | 0.4 |
| *(R)*-Citalopram | 28.9 | ± | 6.1 | 72.8 | ± | 1.4 | 78.0 | ± | 1.4 |
| *(S)*-Citalopram | 37.2 | ± | 1.8 | 80.1 | ± | 0.7 | 77.6 | ± | 0.7 |
| *(R)*-Cycloserine | -0.8 | ± | 3.3 | 17.6 | ± | 7.2 | 28.0 | ± | 7.2 |
| *(S)*-Cycloserine | -2.6 | ± | 1.5 | 0.3 | ± | 2.0 | 35.9 | ± | 2.0 |
| *(R)*-Deprenyl | 21.6 | ± | 1.7 | 50.2 | ± | 2.6 | 62.3 | ± | 2.6 |
| *(S)*-Deprenyl | 26.1 | ± | 0.6 | 58.5 | ± | 2.0 | 68.6 | ± | 2.0 |
| *(E)*-Doxepine | 53.2 | ± | 5.6 | 84.1 | ± | 1.3 | 85.6 | ± | 1.3 |
| *(Z)*-Doxepine | 57.5 | ± | 6.4 | 87.1 | ± | 1.1 | 87.7 | ± | 1.1 |
| *(R)*-Doxylamine | 63.6 | ± | 2.0 | 96.0 | ± | 0.4 | 90.8 | ± | 0.4 |
| *(S)*-Doxylamine | 51.7 | ± | 3.3 | 86.5 | ± | 0.1 | 88.2 | ± | 0.1 |
| *(R)*-Dropropizine | 26.2 | ± | 7.8 | 30.1 | ± | 8.2 | 41.0 | ± | 8.2 |
| *(S)*-Dropropizine | 24.6 | ± | 9.9 | 27.4 | ± | 4.7 | 30.4 | ± | 4.7 |
| *(R)*-Duloxetine | 41.3 | ± | 2.7 | 85.1 | ± | 0.5 | 86.8 | ± | 0.5 |
| *(S)*-Duloxetine | 43.3 | ± | 2.4 | 86.9 | ± | 1.2 | 88.5 | ± | 1.2 |
| *(R,R)*-Ethambutol | 7.7 | ± | 2.1 | | | | 17.9 | ± | 0.0 |
| *(R,S)*-Ethambutol | 4.0 | ± | 0.5 | | | | 10.2 | ± | 0.0 |
| *(S,S)*-Ethambutol | 5.5 | ± | 1.8 | | | | 17.8 | ± | 0.0 |
| *(R)*-Fenfluramine | 38.4 | ± | 1.6 | 73.8 | ± | 5.6 | 86.5 | ± | 5.6 |
| *(S)*-Fenfluramine | 41.9 | ± | 1.6 | 73.9 | ± | 7.5 | 87.1 | ± | 7.5 |
| *(R)*-Fluoxetine | 45.6 | ± | 2.5 | 77.5 | ± | 1.3 | 83.4 | ± | 1.3 |
| *(S)*-Fluoxetine | 47.7 | ± | 3.2 | 78.6 | ± | 0.9 | 82.3 | ± | 0.9 |
| *(R)*-Ibuprofen | 8.3 | ± | 1.6 | 5.6 | ± | 9.6 | -7.2 | ± | 9.6 |
| *(S)*-Ibuprofen | 8.8 | ± | 3.0 | -10.6 | ± | 12.5 | -3.4 | ± | 12.5 |
| *(R)*-Mexiletine | 30.4 | ± | 0.7 | 79.6 | ± | 1.3 | 85.6 | ± | 1.3 |
| *(S)*-Mexiletine | 40.1 | ± | 4.1 | 78.0 | ± | 0.9 | 84.3 | ± | 0.9 |
| *(R)*-Norepinephrine | -4.8 | ± | 5.7 | -19.9 | ± | 20.4 | -1.3 | ± | 20.4 |
| *(S)*-Norepinephrine | -3.3 | ± | 5.7 | 6.1 | ± | 1.0 | 6.6 | ± | 1.0 |
| *(R)*-Oxybutynine | 49.1 | ± | 1.0 | 93.7 | ± | 1.2 | 73.0 | ± | 1.2 |
| *(S)*-Oxybutynine | 59.3 | ± | 0.6 | 96.3 | ± | 0.3 | 83.5 | ± | 0.3 |
| *(R,R)*-Palonosetron | 31.4 | ± | 1.6 | 67.6 | ± | 0.6 | 74.6 | ± | 0.6 |
| *(S,S)*-Palonosetron | 29.9 | ± | 3.0 | 71.6 | ± | 0.7 | 78.0 | ± | 0.7 |
| *(R)*-Phenylephrine | 3.2 | ± | 2.9 | 6.3 | ± | 5.4 | -2.8 | ± | 5.4 |
| *(S)*-Phenylephrine | 5.2 | ± | 0.5 | 6.2 | ± | 3.2 | -5.1 | ± | 3.2 |
| *(R)*-Pramipexole | 19.8 | ± | 2.0 | 51.6 | ± | 2.9 | 55.8 | ± | 2.9 |
| *(S)*-Pramipexole | 12.1 | ± | 1.3 | 34.9 | ± | 1.5 | 35.6 | ± | 1.5 |
| *(R)*-Propafenone | 51.2 | ± | 0.9 | 93.8 | ± | 0.4 | 76.7 | ± | 0.4 |
| *(S)*-Propafenone | 55.0 | ± | 1.7 | 95.9 | ± | 0.4 | 80.2 | ± | 0.4 |
| *(R)*-Propranolol | 44.8 | ± | 2.9 | 79.9 | ± | 0.5 | 84.3 | ± | 0.5 |
| *(S)*-Propranolol | 40.6 | ± | 2.7 | 74.3 | ± | 2.3 | 82.8 | ± | 2.3 |
| Quinine | 15.5 | ± | 0.6 | 54.8 | ± | 1.8 | 38.2 | ± | 1.8 |
| Quinidine | 13.1 | ± | 1.4 | 61.7 | ± | 3.5 | 41.3 | ± | 3.5 |
| *(R)*-Rasagiline | -1.3 | ± | 1.3 | 32.0 | ± | 2.8 | 42.7 | ± | 2.8 |
| *(S)*-Rasagiline | 6.9 | ± | 3.5 | 37.5 | ± | 1.0 | 50.6 | ± | 1.0 |
| *(R)*-Salbutamol | 5.3 | ± | 1.4 | 12.2 | ± | 5.3 | -18.8 | ± | 5.3 |
| *(S)*-Salbutamol | 6.1 | ± | 2.1 | 9.3 | ± | 2.8 | -10.2 | ± | 2.8 |
| *(R)*-Sibutramine | 41.5 | ± | 4.2 | 89.4 | ± | 0.7 | 74.0 | ± | 0.7 |

| | | | | | | | | | |
|---|---|---|---|---|---|---|---|---|---|
| *(S)*-Sibutramine | 38.0 | ± | 0.9 | 87.6 | ± | 0.8 | 72.0 | ± | 0.8 |
| (-)-Sparteine | 45.9 | ± | 1.6 | 85.6 | ± | 2.4 | 82.2 | ± | 2.4 |
| (+)-Sparteine | 43.3 | ± | 0.2 | 83.5 | ± | 3.7 | 81.6 | ± | 3.7 |
| *(R)*-Timolol | 12.9 | ± | 4.0 | 32.1 | ± | 4.6 | 55.0 | ± | 4.6 |
| *(S)*-Timolol | 3.9 | ± | 6.1 | 25.6 | ± | 4.3 | 42.1 | ± | 4.3 |
| *(R)*-Tolterodine | 49.7 | ± | 2.6 | 90.7 | ± | 0.3 | 83.4 | ± | 0.3 |
| *(S)*-Tolterodine | 59.3 | ± | 1.2 | 94.2 | ± | 0.1 | 86.3 | ± | 0.1 |
| *(R)*-Tomoxetine | 49.6 | ± | 1.2 | 89.1 | ± | 0.3 | 84.1 | ± | 0.3 |
| *(S)*-Tomoxetine | 54.4 | ± | 0.9 | 92.6 | ± | 0.2 | 85.9 | ± | 0.2 |
| *(R)*-Venlafaxine | 41.3 | ± | 2.7 | 59.5 | ± | 2.2 | 70.8 | ± | 2.2 |
| *(S)*-Venlafaxine | 43.3 | ± | 2.4 | 57.9 | ± | 0.4 | 66.9 | ± | 0.4 |
| *(R)*-Verapamil | 52.9 | ± | 1.4 | 96.6 | ± | 0.6 | 81.4 | ± | 0.6 |
| *(S)*-Verapamil | 61.4 | ± | 2.2 | 99.4 | ± | 0.3 | 86.1 | ± | 0.3 |
| *(R)*-Zolmitriptan | 1.9 | ± | 2.7 | 21.6 | ± | 3.0 | 43.0 | ± | 3.0 |
| *(S)*-Zolmitriptan | 10.0 | ± | 3.3 | 26.9 | ± | 5.4 | 44.5 | ± | 5.4 |
| **OCT2** | | | | | | | | | |
| **Drug** | **ASP+ inhibition [%] ± SEM** | | | ***(S,S)*-Ethambutol inhibition [%] ± SEM** | | | ***N*-Ethyllidocaine inhibition [%] ± SEM** | | |
| *(R)*-Aclidinium | 107.8 | ± | 6.4 | -0.8 | ± | 11.6 | 69.1 | ± | 1.3 |
| *(S)*-Aclidinium | 101.8 | ± | 8.8 | 2.7 | ± | 9.1 | 57.3 | ± | 4.5 |
| *(R)*-Amisulpride | 65.9 | ± | 1.4 | -5.7 | ± | 5.1 | 11.3 | ± | 3.2 |
| *(S)*-Amisulpride | 38.0 | ± | 2.8 | -13.2 | ± | 2.7 | 1.9 | ± | 5.0 |
| *(R)*-Atenolol | 11.9 | ± | 1.7 | -1.4 | ± | 2.0 | 16.8 | ± | 14.2 |
| *(S)*-Atenolol | 19.1 | ± | 2.3 | 9.0 | ± | 1.7 | 18.8 | ± | 10.3 |
| *(R)*-Bupivacaine | 59.5 | ± | 1.6 | 56.3 | ± | 0.7 | 64.4 | ± | 1.6 |
| *(S)*-Bupivacaine | 43.1 | ± | 2.4 | 38.5 | ± | 1.2 | 50.2 | ± | 0.6 |
| *(R)*-Carvedilol | 99.6 | ± | 3.0 | 13.5 | ± | 6.0 | 58.2 | ± | 2.4 |
| *(S)*-Carvedilol | 94.9 | ± | 3.1 | 8.7 | ± | 12.7 | 48.8 | ± | 2.3 |
| *(R)*-Citalopram | 52.1 | ± | 1.8 | 1.4 | ± | 1.6 | 28.4 | ± | 1.8 |
| *(S)*-Citalopram | 68.6 | ± | 2.1 | -9.7 | ± | 9.1 | 26.5 | ± | 1.4 |
| *(R)*-Cycloserine | 12.3 | ± | 1.1 | 5.0 | ± | 4.2 | 13.6 | ± | 13.9 |
| *(S)*-Cycloserine | 14.6 | ± | 3.5 | 0.8 | ± | 3.0 | 14.6 | ± | 17.1 |
| *(R)*-Deprenyl | 101.0 | ± | 3.3 | 49.1 | ± | 2.1 | 52.3 | ± | 4.6 |
| *(S)*-Deprenyl | 88.0 | ± | 3.6 | 51.6 | ± | 1.1 | 47.7 | ± | 3.4 |
| *(E)*-Doxepine | 109.6 | ± | 7.1 | 82.0 | ± | 1.7 | 95.3 | ± | 1.2 |
| *(Z)*-Doxepine | 108.5 | ± | 6.3 | 56.4 | ± | 4.5 | 82.8 | ± | 3.2 |
| *(R)*-Doxylamine | 73.2 | ± | 2.4 | 26.2 | ± | 5.2 | -13.8 | ± | 9.5 |
| *(S)*-Doxylamine | 64.3 | ± | 3.3 | 26.3 | ± | 1.4 | -17.1 | ± | 10.4 |
| *(R)*-Dropropizine | 40.8 | ± | 4.7 | 28.0 | ± | 14.9 | 8.0 | ± | 5.0 |
| *(S)*-Dropropizine | 51.1 | ± | 4.4 | 20.5 | ± | 10.1 | 7.5 | ± | 3.9 |
| *(R)*-Duloxetine | 53.3 | ± | 2.5 | 33.2 | ± | 3.2 | 50.1 | ± | 0.8 |
| *(S)*-Duloxetine | 70.2 | ± | 3.1 | 19.4 | ± | 4.7 | 46.1 | ± | 1.3 |
| *(R,R)*-Ethambutol | 34.4 | ± | 3.4 | | | | 7.7 | ± | 0.6 |
| *(R,S)*-Ethambutol | 26.0 | ± | 3.2 | | | | 6.8 | ± | 2.5 |
| *(S,S)*-Ethambutol | 38.8 | ± | 7.6 | | | | 10.0 | ± | 0.1 |
| *(R)*-Fenfluramine | 86.5 | ± | 2.6 | 52.8 | ± | 2.9 | 69.0 | ± | 5.9 |
| *(S)*-Fenfluramine | 94.1 | ± | 2.2 | 50.9 | ± | 1.8 | 68.9 | ± | 5.8 |
| *(R)*-Fluoxetine | 65.6 | ± | 3.5 | 28.2 | ± | 1.0 | 48.9 | ± | 2.2 |
| *(S)*-Fluoxetine | 56.6 | ± | 2.6 | 6.4 | ± | 6.5 | 28.7 | ± | 3.8 |
| *(R)*-Ibuprofen | 11.5 | ± | 1.7 | 7.0 | ± | 0.6 | 7.9 | ± | 0.8 |
| *(S)*-Ibuprofen | 10.9 | ± | 0.8 | 14.5 | ± | 5.4 | 10.4 | ± | 0.2 |
| *(R)*-Mexiletine | 47.0 | ± | 1.4 | 52.1 | ± | 4.8 | 52.2 | ± | 4.2 |
| *(S)*-Mexiletine | 52.9 | ± | 1.7 | 58.7 | ± | 3.1 | 51.3 | ± | 6.2 |
| *(R)*-Norepinephrine | 23.1 | ± | 5.6 | -1.8 | ± | 6.3 | 5.4 | ± | 17.3 |
| *(S)*-Norepinephrine | 20.5 | ± | 8.7 | 5.1 | ± | 3.2 | 7.1 | ± | 18.2 |
| *(R)*-Oxybutynine | 86.1 | ± | 1.3 | 7.4 | ± | 7.4 | 28.2 | ± | 6.4 |
| *(S)*-Oxybutynine | 92.8 | ± | 0.7 | 24.5 | ± | 2.3 | 48.6 | ± | 2.0 |
| *(R,R)*-Palonosetron | 89.6 | ± | 3.2 | 30.1 | ± | 2.3 | 49.2 | ± | 0.7 |
| *(S,S)*-Palonosetron | 100.1 | ± | 2.8 | 14.8 | ± | 5.1 | 47.8 | ± | 1.7 |
| *(R)*-Phenylephrine | 8.2 | ± | 2.8 | -8.4 | ± | 1.4 | -1.4 | ± | 3.7 |
| *(S)*-Phenylephrine | 5.4 | ± | 1.3 | 5.5 | ± | 6.6 | -2.7 | ± | 0.5 |

| | | | | | | | | | |
|---|---|---|---|---|---|---|---|---|---|
| *(R)*-Pramipexole | 43.3 | ± | 2.3 | 10.5 | ± | 5.3 | 37.1 | ± | 2.5 |
| *(S)*-Pramipexole | 16.4 | ± | 2.4 | 15.8 | ± | 4.5 | 19.6 | ± | 0.3 |
| *(R)*-Propafenone | 105.5 | ± | 0.4 | 60.0 | ± | 1.4 | 71.4 | ± | 6.3 |
| *(S)*-Propafenone | 104.7 | ± | 0.8 | 55.4 | ± | 3.9 | 81.1 | ± | 2.0 |
| *(R)*-Propranolol | 47.8 | ± | 3.8 | 25.0 | ± | 6.0 | 20.0 | ± | 5.7 |
| *(S)*-Propranolol | 57.3 | ± | 1.7 | 28.4 | ± | 3.8 | 32.4 | ± | 2.3 |
| Quinine | 76.9 | ± | 3.0 | 13.4 | ± | 0.9 | 33.7 | ± | 1.3 |
| Quinidine | 93.4 | ± | 2.1 | 13.3 | ± | 0.7 | 50.8 | ± | 0.6 |
| *(R)*-Rasagiline | 51.7 | ± | 2.8 | 51.3 | ± | 4.7 | 56.7 | ± | 2.7 |
| *(S)*-Rasagiline | 59.6 | ± | 3.6 | 57.0 | ± | 1.9 | 59.5 | ± | 1.6 |
| *(R)*-Salbutamol | 12.8 | ± | 1.5 | -0.3 | ± | 4.2 | -15.0 | ± | 6.8 |
| *(S)*-Salbutamol | -2.8 | ± | 9.0 | 3.0 | ± | 1.4 | -17.4 | ± | 2.3 |
| *(R)*-Sibutramine | 81.6 | ± | 5.1 | 36.0 | ± | 2.3 | 51.2 | ± | 7.4 |
| *(S)*-Sibutramine | 83.9 | ± | 1.1 | 27.8 | ± | 3.8 | 44.0 | ± | 8.1 |
| (-)-Sparteine | 111.0 | ± | 3.6 | 97.3 | ± | 0.7 | 100.5 | ± | 0.3 |
| (+)-Sparteine | 112.4 | ± | 4.4 | 97.5 | ± | 0.4 | 99.9 | ± | 0.3 |
| *(R)*-Timolol | 37.2 | ± | 1.9 | 12.0 | ± | 4.2 | 24.7 | ± | 15.5 |
| *(S)*-Timolol | 50.1 | ± | 4.3 | 14.4 | ± | 3.0 | 25.1 | ± | 13.1 |
| *(R)*-Tolterodine | 105.0 | ± | 1.8 | 91.6 | ± | 0.4 | 98.2 | ± | 0.2 |
| *(S)*-Tolterodine | 108.7 | ± | 2.7 | 88.5 | ± | 6.8 | 99.1 | ± | 0.1 |
| *(R)*-Tomoxetine | 87.1 | ± | 0.4 | 50.2 | ± | 0.8 | 62.5 | ± | 1.5 |
| *(S)*-Tomoxetine | 64.8 | ± | 2.2 | 22.7 | ± | 2.5 | 48.3 | ± | 4.0 |
| *(R)*-Venlafaxine | 29.1 | ± | 3.0 | 18.7 | ± | 1.6 | 23.1 | ± | 4.4 |
| *(S)*-Venlafaxine | 21.8 | ± | 2.8 | 15.2 | ± | 1.7 | 13.4 | ± | 7.2 |
| *(R)*-Verapamil | 95.9 | ± | 5.4 | 27.2 | ± | 8.3 | 44.9 | ± | 12.5 |
| *(S)*-Verapamil | 108.4 | ± | 6.7 | 45.3 | ± | 5.2 | 65.1 | ± | 6.8 |
| *(R)*-Zolmitriptan | 13.2 | ± | 0.5 | 7.7 | ± | 2.1 | -4.7 | ± | 4.5 |
| *(S)*-Zolmitriptan | 17.2 | ± | 1.2 | 13.3 | ± | 6.7 | 7.3 | ± | 5.2 |
| **OCT3** | | | | | | | | | |
| **Drug** | **ASP+ inhibition [%] ± SEM** | | | ***(S,S)*-Ethambutol inhibition [%] ± SEM** | | | ***(S)*-Zolmitriptan inhibition [%] ± SEM** | | |
| *(R)*-Aclidinium | 37.1 | ± | 6.0 | 62.8 | ± | 3.3 | 43.3 | ± | 6.2 |
| *(S)*-Aclidinium | 37.7 | ± | 4.8 | 67.7 | ± | 3.0 | 53.3 | ± | 3.5 |
| *(R)*-Amisulpride | 37.9 | ± | 2.6 | 81.3 | ± | 1.0 | 56.0 | ± | 0.8 |
| *(S)*-Amisulpride | 33.7 | ± | 0.9 | 73.1 | ± | 2.7 | 46.0 | ± | 0.8 |
| *(R)*-Atenolol | 3.9 | ± | 3.9 | 0.9 | ± | 2.4 | 5.5 | ± | 5.0 |
| *(S)*-Atenolol | 0.9 | ± | 3.7 | 2.9 | ± | 2.4 | -3.8 | ± | 8.6 |
| *(R)*-Bupivacaine | 31.4 | ± | 0.7 | 50.3 | ± | 0.4 | 51.8 | ± | 3.3 |
| *(S)*-Bupivacaine | 24.3 | ± | 1.4 | 33.4 | ± | 5.2 | 37.2 | ± | 4.3 |
| *(R)*-Carvedilol | 23.5 | ± | 1.4 | 48.6 | ± | 1.9 | 37.9 | ± | 2.0 |
| *(S)*-Carvedilol | 16.3 | ± | 3.4 | 49.5 | ± | 3.0 | 32.8 | ± | 2.3 |
| *(R)*-Citalopram | 4.5 | ± | 2.0 | 17.2 | ± | 2.4 | 11.8 | ± | 2.8 |
| *(S)*-Citalopram | 10.9 | ± | 0.9 | 31.7 | ± | 3.8 | 19.2 | ± | 4.1 |
| *(R)*-Cycloserine | 17.8 | ± | 9.3 | 7.4 | ± | 6.5 | -0.4 | ± | 7.8 |
| *(S)*-Cycloserine | 19.3 | ± | 9.2 | 12.2 | ± | 3.6 | 6.3 | ± | 1.4 |
| *(R)*-Deprenyl | 12.6 | ± | 1.5 | 24.2 | ± | 2.1 | 18.0 | ± | 4.0 |
| *(S)*-Deprenyl | 9.6 | ± | 1.4 | 20.9 | ± | 3.1 | 11.5 | ± | 5.5 |
| *(E)*-Doxepine | 49.3 | ± | 3.8 | 74.4 | ± | 1.3 | 62.1 | ± | 2.6 |
| *(Z)*-Doxepine | 68.6 | ± | 2.7 | 91.0 | ± | 0.5 | 83.1 | ± | 1.0 |
| *(R)*-Doxylamine | 12.2 | ± | 0.9 | 23.4 | ± | 1.4 | 17.7 | ± | 4.7 |
| *(S)*-Doxylamine | 7.4 | ± | 1.4 | 35.8 | ± | 0.4 | 33.8 | ± | 6.9 |
| *(R)*-Dropropizine | 26.4 | ± | 6.7 | 5.8 | ± | 3.4 | -30.2 | ± | 3.5 |
| *(S)*-Dropropizine | 29.1 | ± | 7.3 | 6.7 | ± | 9.6 | -17.1 | ± | 3.6 |
| *(R)*-Duloxetine | 3.8 | ± | 1.2 | 16.7 | ± | 4.6 | 1.6 | ± | 4.1 |
| *(S)*-Duloxetine | 2.4 | ± | 2.3 | 25.2 | ± | 2.4 | 19.6 | ± | 5.3 |
| *(R,R)*-Ethambutol | 9.1 | ± | 0.3 | | | | -3.7 | ± | 14.8 |
| *(R,S)*-Ethambutol | 2.6 | ± | 3.0 | | | | -8.0 | ± | 15.7 |
| *(S,S)*-Ethambutol | 3.7 | ± | 0.3 | | | | -1.3 | ± | 7.2 |
| *(R)*-Fenfluramine | 7.6 | ± | 5.1 | 40.7 | ± | 0.2 | 55.9 | ± | 2.8 |
| *(S)*-Fenfluramine | 12.7 | ± | 5.6 | 40.7 | ± | 3.7 | 51.9 | ± | 4.8 |
| *(R)*-Fluoxetine | -6.0 | ± | 3.1 | 9.6 | ± | 4.2 | 17.0 | ± | 3.0 |

| | | | | | | | | | |
|---|---|---|---|---|---|---|---|---|---|
| *(S)*-Fluoxetine | -3.1 | ± | 1.0 | 12.3 | ± | 2.0 | 13.8 | ± | 5.1 |
| *(R)*-Ibuprofen | 12.1 | ± | 0.5 | 3.2 | ± | 1.2 | -1.9 | ± | 4.8 |
| *(S)*-Ibuprofen | 12.9 | ± | 2.1 | 11.3 | ± | 3.8 | -0.8 | ± | 3.3 |
| *(R)*-Mexiletine | -17.2 | ± | 3.7 | 11.6 | ± | 1.7 | 25.0 | ± | 4.4 |
| *(S)*-Mexiletine | -6.6 | ± | 4.7 | 11.8 | ± | 3.2 | 30.0 | ± | 7.1 |
| *(R)*-Norepinephrine | 8.4 | ± | 7.4 | 5.7 | ± | 4.4 | -4.3 | ± | 4.1 |
| *(S)*-Norepinephrine | -3.5 | ± | 2.6 | 12.5 | ± | 8.9 | -6.6 | ± | 4.6 |
| *(R)*-Oxybutynine | 31.3 | ± | 3.7 | 64.7 | ± | 5.6 | 42.2 | ± | 2.7 |
| *(S)*-Oxybutynine | 40.5 | ± | 1.5 | 75.7 | ± | 4.9 | 65.2 | ± | 2.2 |
| *(R,R)*-Palonosetron | 21.0 | ± | 0.2 | 68.7 | ± | 1.2 | 62.7 | ± | 1.9 |
| *(S,S)*-Palonosetron | 6.1 | ± | 1.2 | 46.0 | ± | 1.6 | 14.6 | ± | 11.0 |
| *(R)*-Phenylephrine | -4.1 | ± | 1.7 | 5.0 | ± | 3.0 | -2.9 | ± | 6.1 |
| *(S)*-Phenylephrine | 2.7 | ± | 1.5 | 5.6 | ± | 4.3 | -3.9 | ± | 3.5 |
| *(R)*-Pramipexole | 5.3 | ± | 0.7 | 23.1 | ± | 4.4 | 13.0 | ± | 2.8 |
| *(S)*-Pramipexole | 6.1 | ± | 1.5 | 30.2 | ± | 3.6 | 18.4 | ± | 4.5 |
| *(R)*-Propafenone | 17.3 | ± | 6.2 | 71.4 | ± | 2.5 | 53.6 | ± | 2.4 |
| *(S)*-Propafenone | 23.8 | ± | 8.0 | 76.4 | ± | 2.3 | 62.0 | ± | 1.9 |
| *(R)*-Propranolol | -5.7 | ± | 1.6 | 14.6 | ± | 2.0 | -16.8 | ± | 14.2 |
| *(S)*-Propranolol | -2.9 | ± | 1.2 | 32.0 | ± | 1.9 | 38.2 | ± | 4.3 |
| Quinine | 10.3 | ± | 1.3 | 46.2 | ± | 2.1 | 32.1 | ± | 0.9 |
| Quinidine | 38.9 | ± | 3.3 | 85.9 | ± | 0.6 | 67.0 | ± | 4.9 |
| *(R)*-Rasagiline | -11.8 | ± | 3.4 | 13.4 | ± | 3.6 | 10.3 | ± | 4.9 |
| *(S)*-Rasagiline | -7.7 | ± | 3.7 | 8.8 | ± | 2.5 | 6.7 | ± | 5.5 |
| *(R)*-Salbutamol | 10.3 | ± | 2.8 | 19.5 | ± | 4.1 | -6.4 | ± | 2.9 |
| *(S)*-Salbutamol | 1.3 | ± | 1.5 | 14.5 | ± | 4.4 | -3.4 | ± | 2.1 |
| *(R)*-Sibutramine | 2.6 | ± | 1.4 | 35.5 | ± | 3.6 | 38.1 | ± | 4.4 |
| *(S)*-Sibutramine | -0.8 | ± | 0.2 | 23.8 | ± | 2.7 | 24.1 | ± | 7.4 |
| (-)-Sparteine | 0.4 | ± | 4.5 | 20.7 | ± | 1.5 | 11.0 | ± | 8.2 |
| (+)-Sparteine | 5.2 | ± | 6.7 | 32.7 | ± | 2.4 | 28.7 | ± | 7.7 |
| *(R)*-Timolol | 28.6 | ± | 9.7 | 8.2 | ± | 7.9 | 7.6 | ± | 10.9 |
| *(S)*-Timolol | 27.8 | ± | 9.0 | 11.1 | ± | 3.4 | 16.8 | ± | 4.9 |
| *(R)*-Tolterodine | 94.0 | ± | 1.1 | 98.2 | ± | 1.0 | 97.6 | ± | 0.7 |
| *(S)*-Tolterodine | 53.5 | ± | 2.3 | 89.8 | ± | 2.3 | 82.9 | ± | 2.6 |
| *(R)*-Venlafaxine | -2.8 | ± | 0.3 | 12.3 | ± | 5.2 | 12.6 | ± | 2.5 |
| *(R)*-Tomoxetine | 1.1 | ± | 0.5 | 39.0 | ± | 2.9 | 33.2 | ± | 2.3 |
| *(S)*-Tomoxetine | -8.3 | ± | 0.7 | -5.7 | ± | 8.6 | 21.1 | ± | 4.5 |
| *(S)*-Venlafaxine | -11.4 | ± | 0.4 | 4.6 | ± | 1.7 | 21.0 | ± | 3.4 |
| *(R)*-Verapamil | 8.2 | ± | 7.9 | 48.9 | ± | 1.5 | 42.1 | ± | 4.7 |
| *(S)*-Verapamil | 20.8 | ± | 7.7 | 70.7 | ± | 3.3 | 57.6 | ± | 5.4 |
| *(R)*-Zolmitriptan | 27.8 | ± | 8.8 | 6.0 | ± | 6.8 | | | |
| *(S)*-Zolmitriptan | 38.5 | ± | 5.5 | 55.0 | ± | 2.8 | | | |

**Table S5: $IC_{50}$ values of stereoselective OCT inhibiton**

| Transporter | Inhibitor | $IC_{50}$ | 95%-CI | Stereoselectivity |
|---|---|---|---|---|
| OCT1 | *(R)*-Bupivacaine | 10.2 | 6.1 – 17.2 | 1.8-fold for *(S)* |
| | *(S)*-Bupivacaine | 18.7 | 12.0 – 29.0 | |
| | *(R)*-Doxylamine | 22.3 | 12.7 – 39.1 | 1.4-fold for *(S)* |
| | *(S)*-Doxylamine | 32.2 | 7.7 – 135 | |
| | *(R)*-Oxybutynine | 171 | 110 – 264 | 1.2-fold for *(R)* |
| | *(S)*-Oxybutynine | 141 | 103 – 192 | |
| | *(R)*-Pramipexole | 20.2 | 15.8 – 25.8 | 1.6-fold for *(S)* |
| | *(S)*-Pramipexole | 32.2 | 26.0 – 39.9 | |
| OCT2 | *(R)*-Amisulpride | 9.4 | 7.5 – 11.8 | 3.2-fold for *(S)* |
| | *(S)*-Amisulpride | 29.8 | 20.9 – 42.5 | |
| | *(R)*-Bupivacaine | 21.4 | 14.7 – 31.2 | 2.3-fold for *(S)* |
| | *(S)*-Bupivacaine | 48.4 | 37.3 – 62.8 | |
| | *(R)*-Citalopram | 17.8 | 13.2 – 23.9 | 1.6-fold for *(R)* |
| | *(S)*-Citalopram | 10.7 | 7.5 – 15.1 | |
| | *(R)*-Deprenyl | 2.4 | 1.0 – 5.6 | 1.4-fold for *(S)* |
| | *(S)*-Deprenyl | 3.4 | 2.1 – 5.4 | |
| | *(E)*-Doxepine | 0.55 | 0.36 – 0.83 | 4.1-fold for *(Z)* |
| | *(Z)*-Doxepine | 2.26 | 1.61 – 3.17 | |
| | *(R)*-Dropropizine | 80.6 | 35.7 - 182 | 1.4-fold for *(R)* |
| | *(S)*-Dropropizine | 55.6 | 22.9 – 135 | |
| | *(R)*-Duloxetine | 25.3 | 18.4 – 34.8 | 1.5-fold for *(R)* |
| | *(S)*-Duloxetine | 16.6 | 12.2 – 22.5 | |
| | *(R)*-Fenfluramine | 5.2 | 3.7 – 7.3 | 1.7-fold for *(R)* |
| | *(S)*-Fenfluramine | 3.1 | 2.1 – 4.5 | |
| | *(R)*-Oxybutynine | 31.8 | 20.0 – 50.6 | 1.6-fold for *(R)* |
| | *(S)*-Oxybutynine | 20.1 | 14.2 – 28.4 | |
| | *(R,R)*-Palonosetron | 9.4 | 6.0 – 14.9 | 3.2-fold for *(R,R)* |
| | *(S,S)*-Palonosetron | 2.9 | 1.9 – 4.5 | |
| | *(R)*-Pramipexole | 20.4 | 15.9 – 26.0 | 4.0-fold for *(S)* |
| | *(S)*-Pramipexole | 82.4 | 51.1 – 133.1 | |
| | Quinine | 3.8 | 2.9 – 4.9 | 2.9-fold for Quinidine |
| | Quinidine | 11.0 | 8.9 – 13.6 | |
| | *(R)*-Timolol | 121 | 91 – 164 | 2.0-fold for *(R)* |
| | *(S)*-Timolol | 60.2 | 46.0 – 78.8 | |
| | *(R)*-Tolterodine | 0.20 | 0.14 – 0.29 | 1.5-fold for *(R)* |
| | *(S)*-Tolterodine | 0.13 | 0.10 – 0.17 | |
| | *(R)*-Tomoxetine | 6.3 | 4.9 – 8.0 | 2.7-fold for *(S)* |
| | *(S)*-Tomoxetine | 17.1 | 13.2 – 22.3 | |
| | *(R)*-Verapamil | 8.2 | 7.1 – 9.6 | 1.8-fold for *(R)* |
| | *(S)*-Verapamil | 4.6 | 3.7 – 5.7 | |
| OCT3 | *(E)*-Doxepine | 33.0 | 22.1 – 49.1 | 2.2-fold for *(E)* |
| | *(Z)*-Doxepine | 14.9 | 10.2 – 21.6 | |
| | *(R)*-Oxybutynine | 65.0 | 50.1 – 84.2 | 1.6-fold for *(R)* |
| | *(S)*-Oxybutynine | 40.0 | 31.9 – 50.3 | |
| | *(R,R)*-Palonosetron | 10.7 | 9.3 – 12.4 | 2.5-fold for *(R,R)* |
| | *(S,S)*-Palonosetron | 27.0 | 22.0 – 33.1 | |

| | | | | |
|---|---|---|---|---|
| | *(R)*-Propranolol | 195 | 146 – 260 | 2.5-fold for *(R)* |
| | *(S)*-Propranolol | 69 | 58 – 81 | |
| | Quinine | 78.9 | 48.1 – 130 | 3.2-fold for Quinidine |
| | Quinidine | 249 | 159 – 390 | |
| | *(R)*-Tolterodine | 2.3 | 1.8 – 2.9 | 11-fold for *(S)* |
| | *(S)*-Tolterodine | 25.3 | 19.6 – 32.1 | |
| | *(R)*-Verapamil | 29.7 | 21.4 – 41.3 | 2.2-fold for *(R)* |
| | *(S)*-Verapamil | 13.5 | 10.5 – 17.3 | |
| | *(R)*-Zolmitriptan | 440 | 80 – 2425 | 25-fold for *(R)* |
| | *(S)*-Zolmitriptan | 17.3 | 12.3 – 24.4 | |

**Table S6: $IC_{50}$ values of stereoselective MAT inhibition**

| Transporter | Inhibitor | $IC_{50}$ [µM] | 95%-CI | Stereoselectivity |
|---|---|---|---|---|
| NET | *(E)*-Doxepine | 0.69 | 0.51 – 0.94 | 3.90-fold for *(Z)* |
| | *(Z)*-Doxepine | 2.69 | 1.94 – 3.79 | |
| | *(R)*-Duloxetine | 0.28 | 0.18 – 0.44 | 1.11-fold for *(S)* |
| | *(S)*-Duloxetine | 0.31 | 0.18 – 0.52 | |
| | *(R)*-Fenfluramine | 81.7 | 57.5 – 116.2 | 4.75-fold for *(R)* |
| | *(S)*-Fenfluramine | 17.2 | 12.9 – 22.8 | |
| | *(R)*-Sibutramine | 22.7 | 4.7 – 109.3 | 3.90-fold for *(S)* |
| | *(S)*-Sibutramine | 88.5 | 0.38 - 20450 | |
| | *(R)*-Tomoxetine | 0.09 | 0.0001 – 62.9 | 1.33-fold for *(S)* |
| | *(S)*-Tomoxetine | 0.12 | 0.09 – 0.18 | |
| | *(R)*-Venlafaxine | 0.97 | 0.38 – 2.44 | 8.74-fold for *(S)* |
| | *(S)*-Venlafaxine | 8.48 | 1.80 – 39.9 | |
| SERT | *(R)*-Citalopram | 0.196 | 0.132 – 0.292 | 6.33-fold for *(R)* |
| | *(S)*-Citalopram | 0.031 | 0.021 – 0.045 | |
| | *(E)*-Doxepine | 0.55 | 0.38 – 0.80 | 3.27-fold for *(Z)* |
| | *(Z)*-Doxepine | 1.80 | 1.21 – 2.68 | |
| | *(R)*-Duloxetine | 0.035 | 0.019 – 0.062 | 1.12-fold for *(R)* |
| | *(S)*-Duloxetine | 0.031 | 0.019 – 0.049 | |
| | *(R)*-Fenfluramine | 4.94 | 3.21 – 7.59 | 6.10-fold for *(R)* |
| | *(S)*-Fenfluramine | 0.81 | 0.51 – 1.29 | |
| | *(R)*-Fluoxetine | 0.11 | 0.09 – 0.14 | 1.07-fold for *(R)* |
| | *(S)*-Fluoxetine | 0.11 | 0.08 – 0.14 | |
| | *(R)*-Sibutramine | 54.5 | 28.8 – 103.0 | 1.09-fold for *(R)* |
| | *(S)*-Sibutramine | 59.4 | 33.1 – 106.7 | |
| | *(R)*-Tomoxetine | 0.14 | 0.11 – 0.18 | 1.08-fold for *(R)* |
| | *(S)*-Tomoxetine | 0.13 | 0.10 – 0.17 | |
| | *(R)*-Venlafaxine | 0.070 | 0.046 – 0.105 | 1.89-fold for *(R)* |
| | *(S)*-Venlafaxine | 0.037 | 0.031 – 0.043 | |

**> Organic cation transporter 1 (OCT1/*SLC22A1*)**

MPTVDDILEQVGESGWFQKQAFLILCLLSAAFAPICVGIVFLGFTPDHHCQSPGVAELSQRCGWSPAEELNYTVPGLGPAGEA
FLGQCRRYEVDWNQSALSCVDPLASLATNRSHLPLGPCQDGWVYDTPGSSIVTEFNLVCADSWKLDLFQSCLNAGFLFGSLGV
GYFADRFGRKLCLLGTVLVNAVSGVLMAFSPNYMSMLLFRLLQGLVSKGNWMAGYTLITEFVGSGSRRTVAIMYQMAFTVGLV
ALTGLAYALPHWRWLQLAVSLPTFLFLLYYWCVPESPRWLLSQKRNTEAIKIMDHIAQKNGKLPPADLKMLSLEEDVTEKLSP
SFADLFRTPRLRKRTFILMYLWFTDSVLYQGLILHMGATSGNLYLDFLYSALVEIPGAFIALITIDRVGRIYPMAMSNLLAGA
ACLVMIFISPDLHWLNIIIMCVGRMGITIAIQMICLVNAELYPTFVRNLGVMVCSSLCDIGGIITPFIVFRLREVWQALPLIL
FAVLGLLAAGVTLLLPETKGVALPETMKDAENLGRKAKPKENTIYLKVQTSEPSGT

**> Organic cation transporter 2 (OCT2/*SLC22A2*)**

MPTTVDDVLEHGGEFHFFQKQMFFLLALLSATFAPIYVGIVFLGFTPDHRCRSPGVAELSLRCGWSPAEELNYTVPGPGPAGE
ASPRQCRRYEVDWNQSTFDCVDPLASLDTNRSRLPLGPCRDGWVYETPGSSIVTEFNLVCANSWMLDLFQSSVNVGFFIGSMS
IGYIADRFGRKLCLLTTVLINAAAGVLMAISPTYTWMLIFRLIQGLVSKAGWLIGYILITEFVGRRYRRTVGIFYQVAYTVGL
LVLAGVAYALPHWRWLQFTVALPNFFFLLYYWCIPESPRWLISQNKNAEAMRIIKHIAKKNGKSLPASLQRLRLEEETGKKLN
PSFLDLVRTPQIRKHTMILMYNWFTSSVLYQGLIMHMGLAGDNIYLDFFYSALVEFPAAFMIILTIDRIGRRYPWAASNMVAG
AACLASVFIPGDLQWLKIIISCLGRMGITMAYEIVCLVNAELYPTFIRNLGVHICSSMCDIGGIITPFLVYRLTNIWLELPLM
VFGVLGLVAGGLVLLLPETKGKALPETIEEAENMQRPRKNKEKMIYLQVQKLDIPLN

**> Organic cation transporter 3 (OCT1/*SLC22A3*)**

MPSFDEALQRVGEFGRFQRRVFLLLCLTGVTLAFLFVGVVFLGTQPDHYWCRGPSAAALAERCGWSPEEEWNRTAPASRGPGP
PERRGRCQRYLLEAANDSASATSALSCADPLAAFPNRSAPLVPCRGGWRYAQAHSTIVSEFDLVCVNAWMLDLTQAILNLGFL
TGAFTLGYAADRYGRIVIYLLSCLGVGVTGVVVAFAPNFPVFVIFRFLQGVFGKGTWMTCYVIVTEIVGSKQRRIVGIVIQMF
FTLGIIILPGIAYFIPNWQGIQLAITLPSFLFLLYYWVVPESPRWLITRKKGDKALQILRRIAKCNGKYLSSNYSEITVTDEE
VSNPSFLDLVRTPQMRKCTLILMFAWFTSAVVYQGLVMRLGIIGGNLYIDFFISGVVELPGALLILLTIERLGRRLPFAASNI
VAGVACLVTAFLPEGIAWLRTTVATLGRLGITMAFEIVYLVNSELYPTTLRNFGVSLCSGLCDFGGIIAPFLLFRLAAVWLEL
PLIIFGILASICGGLVMLLPETKGIALPETVDDVEKLGSPHSCKCGRNKKTPVSRSHL

**> Norepinephrine transporter (NET/*SLC6A2*)**

MLLARMNPQVQPENNGADTGPEQPLRARKTAELLVVKERNGVQCLLAPRDGDAQPRETWGKKIDFLLSVVGFAVDLANVWRFP
YLCYKNGGGAFLIPYTLFLIIAGMPLFYMELALGQYNREGAATVWKICPFFKGVGYAVILIALYVGFYYNVIIAWSLYYLFSS
FTLNLPWTDCGHTWNSPNCTDPKLLNGSVLGNHTKYSKYKFTPAAEFYERGVLHLHESSGIHDIGLPQWQLLLCLMVVVIVLY
FSLWKGVKTSGKVVWITATLPYFVLFVLLVHGVTLPGASNGINAYLHIDFYRLKEATVWIDAATQIFFSLGAGFGVLIAFASY
NKFDNNCYRDALLTSSINCITSFVSGFAIFSILGYMAHEHKVNIEDVATEGAGLVFILYPEAISTLSGSTFWAVVFFVMLLAL
GLDSSMGGMEAVITGLADDFQVLKRHRKLFTFGVTFSTFLLALFCITKGGIYVLTLLDTFAAGTSILFAVLMEAIGVSWFYGV
DRFSNDIQQMMGFRPGLYWRLCWKFVSPAFLLFVVVVSIINFKPLTYDDYIFPPWANWVGWGIALSSMVLVPIYVIYKFLSTQ
GSLWERLAYGITPENEHHLVAQRDIRQFQLQHWLAI

**> Dopamine transporter (DAT/*SLC6A3*)**

MSKSKCSVGLMSSVVAPAKEPNAVGPKEVELILVKEQNGVQLTSSTLTNPRQSPVEAQDRETWGKKIDFLLSVIGFAVDLANV
WRFPYLCYKNGGGAFLVPYLLFMVIAGMPLFYMELALGQFNREGAAGVWKICPILKGVGFTVILISLYVGFFYNVIIAWALHY
LFSSFTTELPWIHCNNSWNSPNCSDAHPGDSSGDSSGLNDTFGTTPAAEYFERGVLHLHQSHGIDDLGPPRWQLTACLVLVIV
LLYFSLWKGVKTSGKVVWITATMPYVVLTALLLRGVTLPGAIDGIRAYLSVDFYRLCEASVWIDAATQVCFSLGVGFGVLIAF
SSYNKFTNNCYRDAIVTTSINSLTSFSSGFVVFSFLGYMAQKHSVPIGDVAKDGPGLIFIIYPEAIATLPLSSAWAVVFFIML
LTLGIDSAMGGMESVITGLIDEFQLLHRHRELFTLFIVLATFLLSLFCVTNGGIYVFTLLDHFAAGTSILFGVLIEAIGVAWF
YGVGQFSDDIQQMTGQRPSLYWRLCWKLVSPCFLLFVVVVSIVTFRPPHYGAYIFPDWANALGWVIATSSMAMVPIYAAYKFC
SLPGSFREKLAYAIAPEKDRELVDRGEVRQFTLRHWLKV

**> Serotonin transporter (SERT/*SLC6A4*)**

METTPLNSQKQLSACEDGEDCQENGVLQKVVPTPGDKVESGQISNGYSAVPSPGAGDDTRHSIPATTTTLVAELHQGERETWG
KKVDFLLSVIGYAVDLGNVWRFPYICYQNGGGAFLLPYTIMAIFGGIPLFYMELALGQYHRNGCISIWRKICPIFKGIGYAIC
IIAFYIASYYNTIMAWALYYLISSFTDQLPWTSCKNSWNTGNCTNYFSEDNITWTLHSTSPAEEFYTRHVLQIHRSKGLQDLG
GISWQLALCIMLIFTVIYFSIWKGVKTSGKVVWVTATFPYIILSVLLVRGATLPGAWRGVLFYLKPNWQKLLETGVWIDAAAQ
IFFSLGPGFGVLLAFASYNKFNNNCYQDALVTSVVNCMTSFVSGFVIFTVLGYMAEMRNEDVSEVAKDAGPSLLFITYAEAIA
NMPASTFFAIIFFLMLITLGLDSTFAGLEGVITAVLDEFPHVWAKRRERFVLAVVITCFFGSLVTLTFGGAYVVKLLEEYATG
PAVLTVALIEAVAVSWFYGITQFCRDVKEMLGFSPGWFWRICWVAISPLFLLFIICSFLMSPPQLRLFQYNYPYWSIILGYCI
GTSSFICIPTYIAYRLIITPGTFKERIIKSITPETPTEIPCGDIRLNAV

**Figure 1: Amino acid sequences of overexpressed OCTs and MATs**

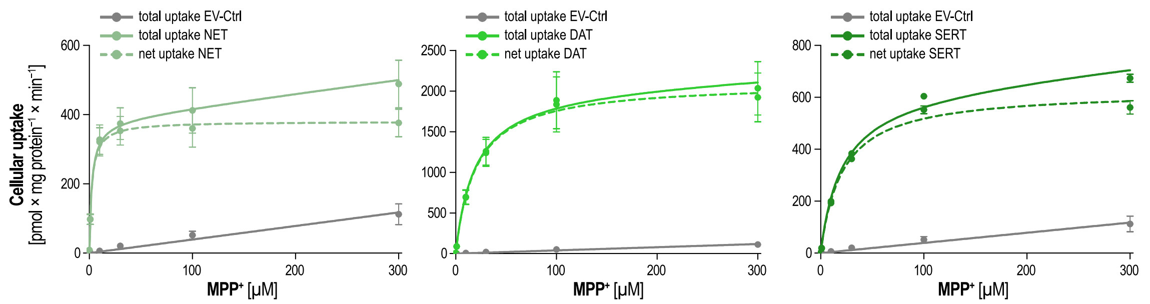


**Figure S2: MPP⁺ transport by monoamine transporters.** NET, DAT and SERT overexpressing as well as empty-vector transfected HEK293 cells were incubated with increasing concentrations of MPP⁺ for 2 min. Subsequently, cells were washed and lysed before intracellular MPP⁺ was quantified by LC-MS/MS analysis. Data is presented as mean ± SEM of three independent experiments.

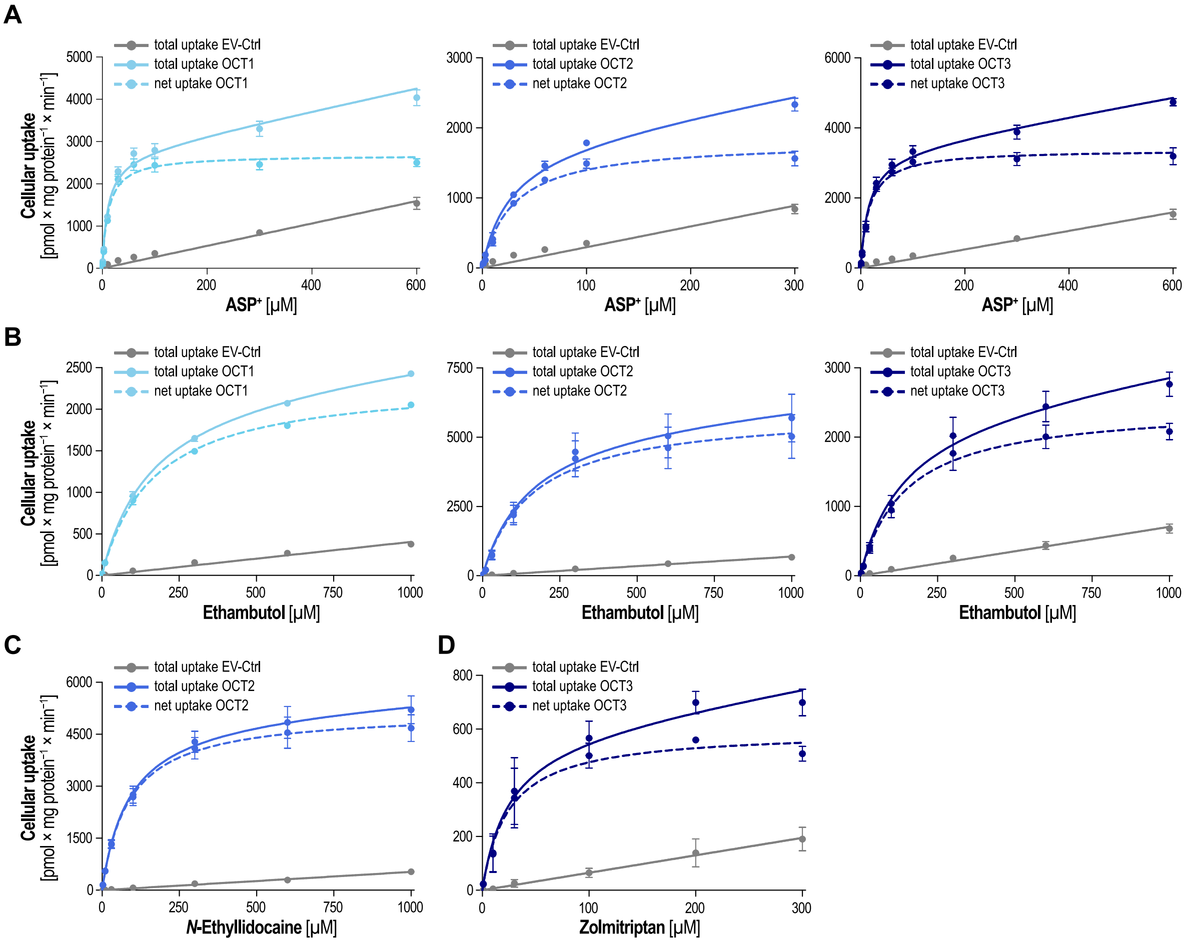


**Figure S3: Novel transport data for OCT model substrates.** OCT1, OCT2, and OCT3 overexpressing as well as empty-vector transfected HEK293 cells were incubated with increasing concentrations of ASP⁺ (A), ethambutol (B), N-ethyllidocaine (C) or zolmitriptan (D) for 2 min. Subsequently, cells were washed and lysed before intracellular drug concentrations were quantified by LC-MS/MS analysis. Data is presented as mean ± SEM of three independent experiments.

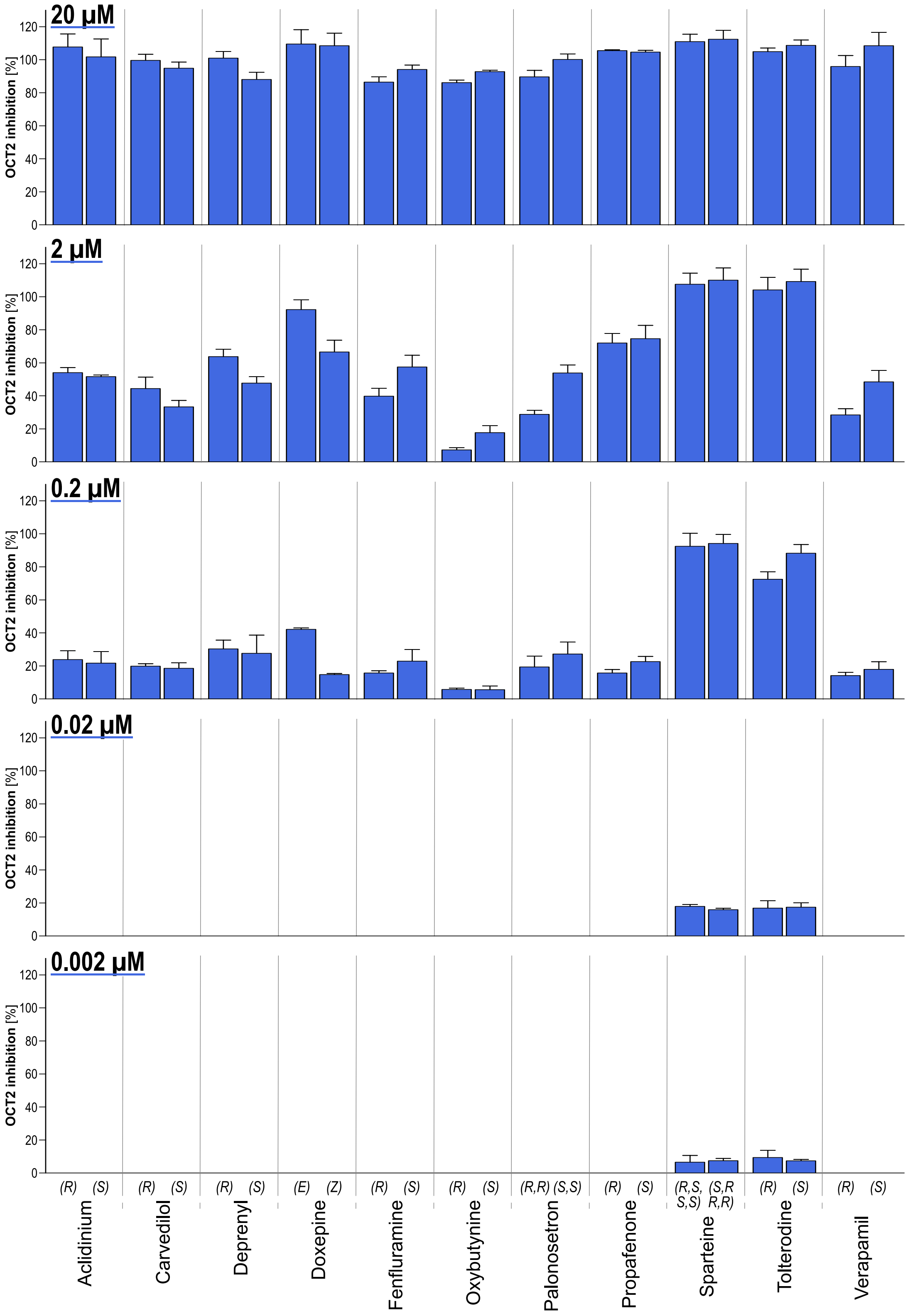


**Figure S4: Inhibition screening of OCT2 ASP⁺ uptake with reduced inhibitor concentrations.** OCT2 overexpressing HEK293 cells and empty-vector transfected controls were incubated with 2 µM ASP⁺ and 0.002 – 20 µM inhibitor for 5 min. Concentrations of 0.02 and 0.002 µM inhibitor were only tested for tolterodine and sparteine enantiomers.

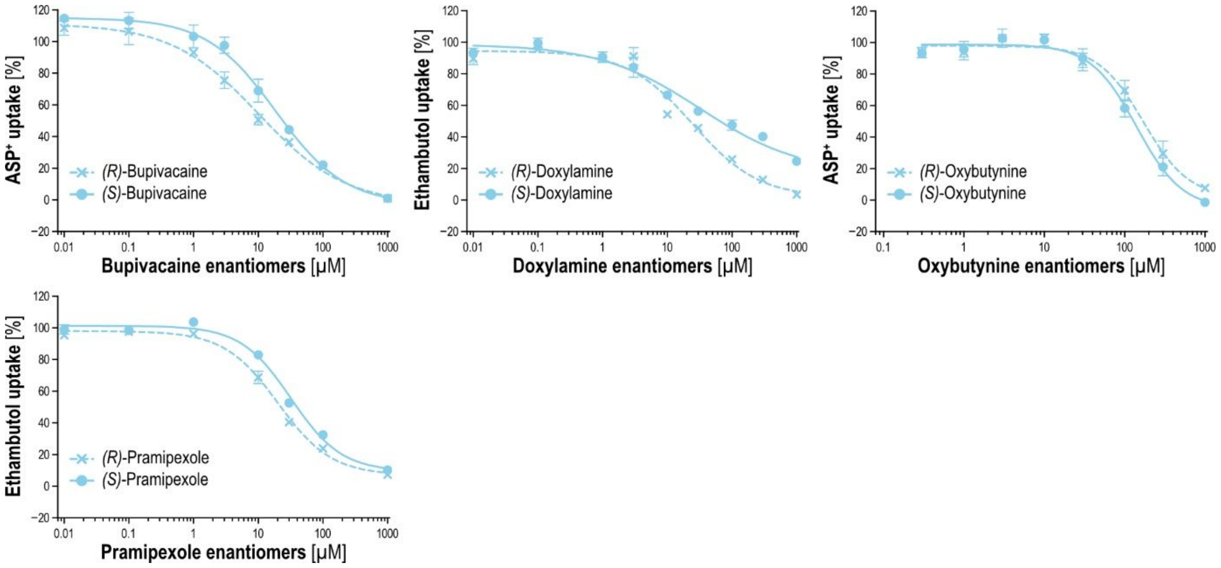


**Figure S5: Stereoselective concentration-dependent OCT1 inhibition.** Data is presented as mean ± SEM of three independent experiments. Model substrate concentration was 2 µM. The used model substrate, either ASP+ or ethambutol, is indicated on the vertical axis.

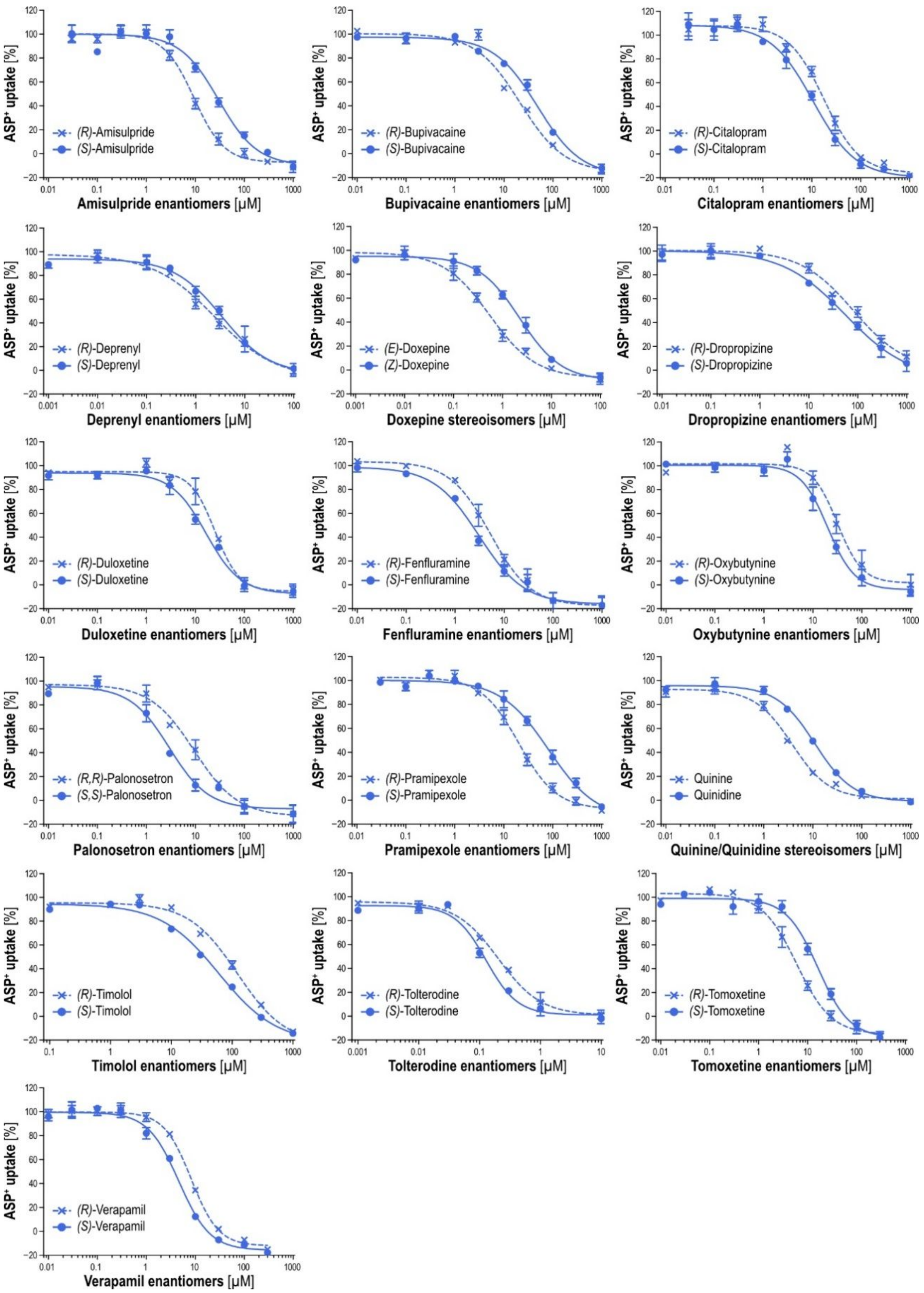


**Figure S6: Stereoselective concentration-dependent OCT2 inhibition.** Data is presented as mean ± SEM of three independent experiments. $ASP^+$ concentration was 2 µM.

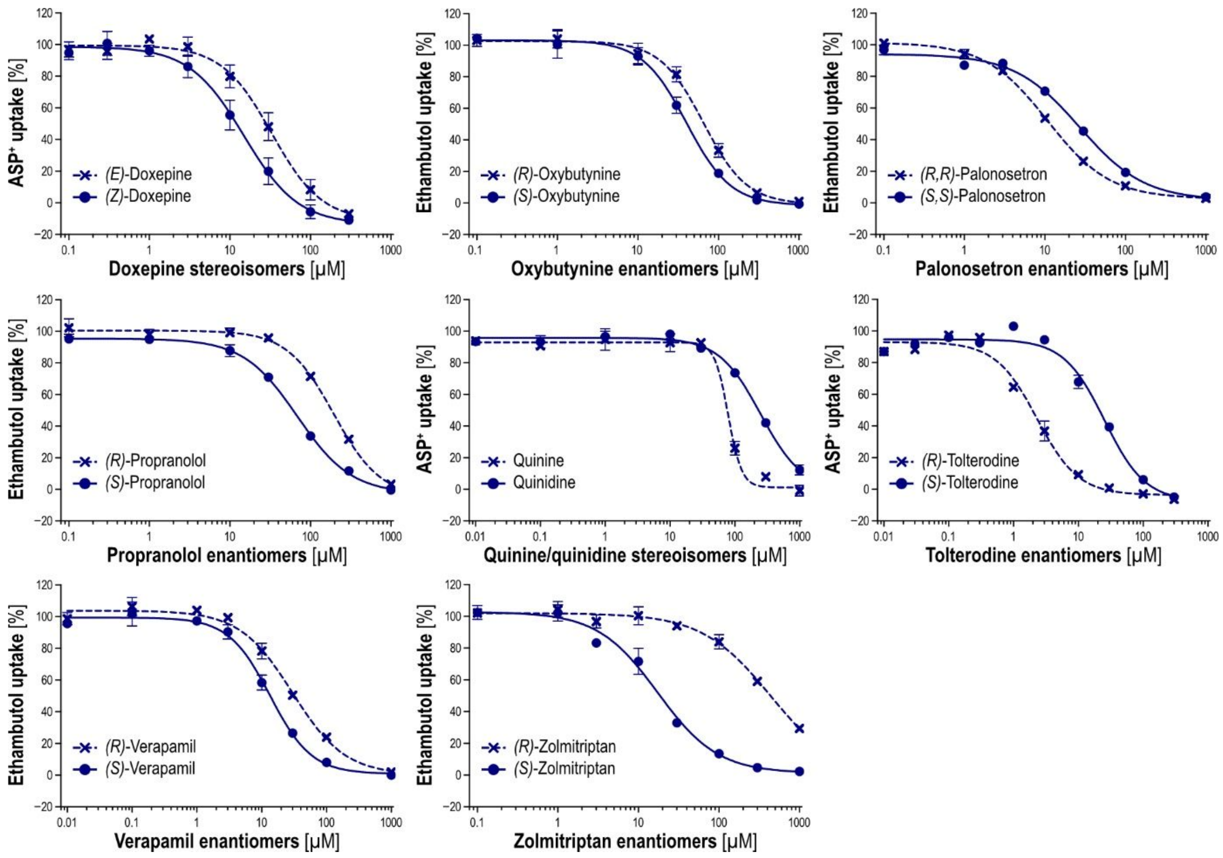


**Figure S7: Stereoselective concentration-dependent OCT3 inhibition.** Data is presented as mean ± SEM of three independent experiments. Model substrate concentration was 2 µM. The used model substrate, either ASP+ or ethambutol, is indicated on the vertical axis.
